# Diketopyrrolopyrrole‐Based Cation as a Semiconducting Spacer in Layered Perovskite

**DOI:** 10.1002/adma.74681

**Published:** 2026-08-18

**Authors:** Waygen Thor, Colin Jeanguenat, Louise De Cian, Demetra Tsokkou, Parnian Ferdowsi, Nicolas J. Diercks, Natalie Banerji, Jun‐Ho Yum, Kevin Sivula

**Affiliations:** ^1^ Laboratory For Molecular Engineering of Optoelectronic Nanomaterials (LIMNO), Institut Des Sciences et Ingénierie Chimiques (ISIC) École Polytechnique Fédérale de Lausanne (EPFL) Lausanne Switzerland; ^2^ FemtoMat Research Group, Department Für Chemie Biochemie und Pharmazie, University of Bern Bern Switzerland

**Keywords:** 2D perovskite, electron transfer, energy conversion efficiency, layered structure, materials science, optoelectronics, organic semiconductor, organic‐inorganic hybrid materials, perovskite, ultrafast laser spectroscopy

## Abstract

Incorporating π‐conjugated organic spacers in layered “2D” perovskites in order to extend solar light harvesting has remained a challenge, as typically‐incorporated spacers (e.g. phenethylammonium, PEA^+^) do not absorb deep into the visible spectrum. Here, we introduce a visible‐light‐absorbing π‐conjugated dithiophene–diketopyrrolopyrrole‐based spacer cation that forms a pure iodide layered perovskite that exhibits complementary organic–inorganic absorption and a type‐II nano‐heterojunction electronic structure. By chain length engineering, a dihexyl‐substituted diketopyrrolopyrrole cation (DPP‐dH^2+^) is identified as the optimal length required to form an ordered layered structure **(DPP‐dH)PbI_4_**. Transient absorption spectroscopy reveals bidirectional charge transfer, with hole transfer from the inorganic [PbI_4_]^2−^ slabs to the organic spacer, and electron transfer in the reverse direction upon excitation at longer wavelengths. Time‐resolved microwave conductivity and space‐charge‐limited current measurements demonstrate reduced trap density and electron mobilities up to 1.3 × 10^−3^ cm^2^V^−1^s^−1^. Leveraging its extended visible absorption, the resulting layered perovskite exhibits a marked improvement in photovoltaic power conversion efficiency compared to **(PEA)_2_PbI_4_** as well as an extended incident photon harvesting reaching 650 nm (1.9 eV), establishing diketopyrrolopyrrole spacers as a promising platform for next‐generation optoelectronics.

## Graphical abstract

Diketopyrrolopyrrole cation incorporated layered perovskites exhibiting extended absorption profiles and enhanced charge carrier mobility with type‐II energy alignment. The improved photoconversion efficiency demonstrates the critical role of incorporating organic semiconductors in optimizing layered perovskite performance.

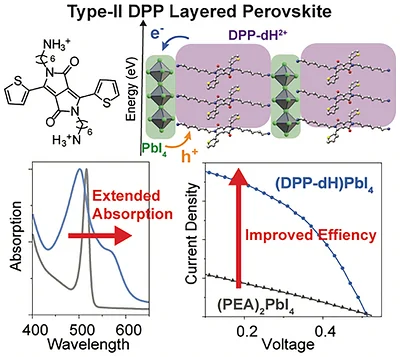

## 1 Introduction

Layered two‐dimensional hybrid organic–inorganic (or 2D) perovskites have emerged as attractive alternatives to conventional three‐dimensional (3D) perovskites, owing to their suppressed ion migration and trap‐state densities [1, 2], enhanced environmental stability [3, 4], and highly tunable structural and electronic properties [5, 6, 7, 8]. These advantages have motivated growing interest in layered perovskites for applications ranging from photovoltaics [9, 10], transistors [11, 12], and other optoelectronic devices [13, 14, 15, 16]. However, the typically‐employed organic spacers are electronically insulating and thus their placement as layers between the inorganic octahedral slabs inherently limits out‐of‐plane charge‐carrier transport, resulting in a highly bound excitonic nature of charge carriers and overall lower device performance compared to 3D perovskites [17, 18, 19]. In addition, quantum confinement effects lead to widened bandgaps, restricting light absorption primarily to the near‐visible region [3, 20, 21], which further limits the application of layered perovskites for efficient solar energy conversion.

Although increasing the number of adjacent inorganic slabs in the so‐called quasi‐2D perovskites can offer some compromise on the optoelectronic properties [22, 23, 24], the resulting materials often suffer from phase heterogeneity, reduced environmental stability, and inefficient energy transfer processes. Alternatively, incorporating π‐conjugated semiconducting organic spacer cations into layered hybrid organic–inorganic perovskites has emerged as a powerful approach to broaden their optoelectronic functionality while maintaining phase purity and environmental stability. Early studies demonstrated that carbazole‐based spacers can enhance the electrical conductivity of layered perovskites by up to three orders of magnitude compared to conventional insulating spacers, highlighting the potential of electronically active organic layers [25]. Moreover, π‐conjugated organic ligands can enable charge transfer between the organic and inorganic layers, forming type‐II nano‐heterojunction architectures that facilitate efficient charge separation and transport [26, 27, 28]. Molecular engineering of conjugated oligothiophene spacers established that tuning the molecular backbone provides an effective route to modulate the electronic structure and energy‐level alignment of layered perovskites [5]. More recently, pyrene‐based spacers with systematically varied alkyl chain lengths were shown to enable control over interfacial energy alignment and charge‐transfer characteristics [29], while biphenyl‐thiophene spacers were demonstrated to suppress ion migration, reduce defect densities, and improve radiative recombination efficiency [30]. Previous examples employing naphthalene diimide (NDI)‐based spacers demonstrated that these semiconducting ligands can be successfully integrated into perovskite structures with favorable band alignment [31, 32, 33], leading to enhanced electron mobility and charge carrier lifetime, which are beneficial for optoelectronic applications. Furthermore, it has been directly shown that light absorption by organic spacers can generate free charge carriers in layered perovskites [34]. This finding suggests that by integrating appropriately tuned spacer cations, layered perovskite systems can move beyond exciton‐bound behavior toward direct free‐carrier generation and transport in separate conduction channels (e.g., holes in the organic and electrons in the inorganic slabs).

Despite the promise of this approach, most implementations remain limited to π‐conjugated spacers with absorption in the ultraviolet or blue regions, and extending spacer absorption deeper into the visible spectrum has remained a significant challenge. Although a naphthalene–iminoimide cation with absorption extending into the visible light region (up to 550 nm, 2.25 eV) has recently been reported, higher‐band gap bromide‐containing mixed halide perovskite layers were required to optimize nano‐heterojunction band alignment [34]. Mixed halide perovskites are reported to suffer from halide segregation, a phenomenon known to increase trap densities in materials incorporating mixed halides [35, 36, 37]. Therefore, demonstrations of π‐conjugated spacers that simultaneously extend visible‐light absorption and establish efficient organic–inorganic charge separation in a pure *n* = 1 layered iodide perovskite remain rare, underscoring the need for new spacer designs that combine deep visible absorption with efficient charge separation in phase‐pure iodide layered perovskites.

To address this, the π‐conjugated diketopyrrolopyrrole (DPP) core molecule represents an especially compelling candidate. DPP derivatives are widely recognized in organic electronics for their exceptionally high absorptivity and tunable absorption profiles extending deep into the visible region, arising from their intrinsically small energy gap [38, 39, 40, 41]. Equally important, the rigid and planar architecture of the DPP core promotes strong π–π interactions [42, 43], a structural characteristic expected to support the formation of well‐ordered and potentially more stable layered perovskite architectures [44, 45, 46]. These attributes collectively position DPP‐based spacers as an attractive platform for expanding both the optical functionality and structural robustness of layered hybrid perovskites. Although DPP is an attractive π‐conjugated semiconductor for incorporation into layered hybrid perovskites, convincing experimental evidence for the formation of a DPP incorporated layered perovskite remains limited [47].

In this work, we introduce a visible‐light‐absorbing spacer cation based on the dithiophene‐DPP core and evaluate its ability to form layered perovskites. Through tuning of the alkyl linker length, we demonstrate that the dihexyl‐substituted DPP can be successfully incorporated to form a layered perovskite structure, **(DPP‐dH)PbI_4_**. The resulting DPP‐based layered perovskite exhibits complementary absorption from both the inorganic perovskite slabs and the organic chromophore, enabling complementary charge transfer and promoting efficient charge separation through dual excitation pathways.

## 2 Results and Discussions

To evaluate the feasibility of incorporating DPP‐based organic spacers into lead iodide layered perovskites, ammonium‐terminated alkyl chains with various lengths were attached to the dithiophene‐DPP core via N‐alkylation (see Scheme S1). Iodide salts of the resulting ammonium cations were tested for perovskite film formation. All thin films were fabricated under identical conditions using a solution‐processed two‐step spin‐coating procedure (see experimental details, Supporting Information for full details). Briefly, the organic spacer and PbI_2_ were mixed in a 1:1 molar ratio in DMF, chlorobenzene was applied as an antisolvent during the second spin step, and the films were annealed at 160 °C for 1 h. UV–vis absorption spectra and XRD diffractograms show that spacers with dipropyl and dibutyl ammonium functionalization give rise to a 1D excitonic feature with predominantly amorphous diffraction patterns (Figure S1). In contrast, the dihexyl derivative (coded as DPP‐dH^2+^, see structure in Figure 1a) produces a well‐defined layered perovskite phase, shown schematically in Figure 1b as supported by the diffraction pattern (Figure 1c) and the emergence of the layered‐perovskite excitonic peak at *λ* = 510 nm in the absorption spectrum that is absent in the pure ligand film (Figure 1d). We note that the dependence of the alkyl chain length on the formation of the layered phase aligns with previous reports on NDI‐based systems, where shorter linkers form 1D structures [48] and six‐carbon linkers afford layered perovskite formation [32]. To form an ordered layered structure, not only is efficient packing of the DPP‐core required, but the positioning of the terminal diammonium groups is also critical for establishing electrostatic and hydrogen‐bonding interactions with the iodide of the inorganic perovskite slab. The shorter dipropyl and dibutyl linkers appear to be insufficient to satisfy these requirements, resulting in the formation of amorphous structures. In contrast, the greater length and conformational flexibility of the dihexyl linker reasonably allow the diammonium terminal group to interact favorably with the inorganic slab and simultaneously enable efficient packing of the DPP cores. Successful formation of the layered phase when using the dihexyl derivative is further supported by the grazing‐incidence wide‐angle x‐ray scattering (GIWAXS) pattern (Figure 1c, inset), which exhibits diffraction rings with increased scattering intensity in the *q_z_* direction characteristic of layered perovskites with slabs oriented preferentially parallel with the substrate [49, 50].

**FIGURE 1:**
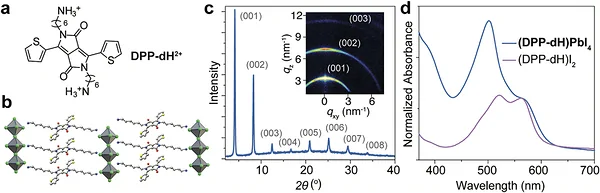
(a) Chemical structure of the divalent dithiophene‐DPP spacer investigated herein. (b) Illustration of the incorporation of DPP‐dH^2+^ with the [PbI_4_]^2−^ slabs, forming a layered perovskite structure. (c) XRD pattern and corresponding uncorrected GIWAXS patterns (inset) of **(DPP‐dH)PbI_4_** measured with Cu Kα_1_ x‐ray source. (d) The UV–vis absorption spectrum of **(DPP‐dH)PbI_4_** layered perovskite compared with that of (DPP‐dH)I_2_ film.

The electron energy level alignment of the organic and inorganic components of **(DPP‐dH)PbI_4_** was estimated by ultraviolet photoelectron spectroscopy (UPS) measurements (Figure S2) using a comparison to (DPP‐dH)I_2_. The relative energy levels of DPP‐dH^2+^ and the inorganic perovskite [PbI_4_]^2−^ slab (Figure 2a) suggest the formation of a type‐II layered perovskite with possible bidirectional charge transfer. This electron level alignment implies a complementary charge transfer mechanism: holes transfer from the inorganic slab to the organic spacer upon excitation of the perovskite, whereas electrons transfer from the organic spacer to the inorganic slab upon excitation of the DPP‐dH cation. It is worth noting that the energy levels of the DPP‐dH^2+^ cation were measured from neat (DPP‐dH)I_2_ films and may therefore differ from those in the layered perovskite due to polarization and interfacial dipole effect by the adjacent [PbI_4_]^2−^ slabs. Additional experimental data are needed to confirm a type‐II heterojunction structure with bidirectional charge transfer. The quenched photoluminescence of **(DPP‐dH)PbI_4_** compared to that of **(PEA)_2_PbI_4_** and the (DPP‐dH)I_2_ films supports the formation of a type‐II nano‐heterojunction that promotes interfacial charge separation, thereby suppressing radiative recombination (Figure S3). Consistent with this, although ligand emission remains observable, it is most reasonably attributed to direct excitation of the DPP layer at the excitation wavelength rather than Förster energy transfer from the inorganic [PbI_4_]^2−^ slabs.

**FIGURE 2:**
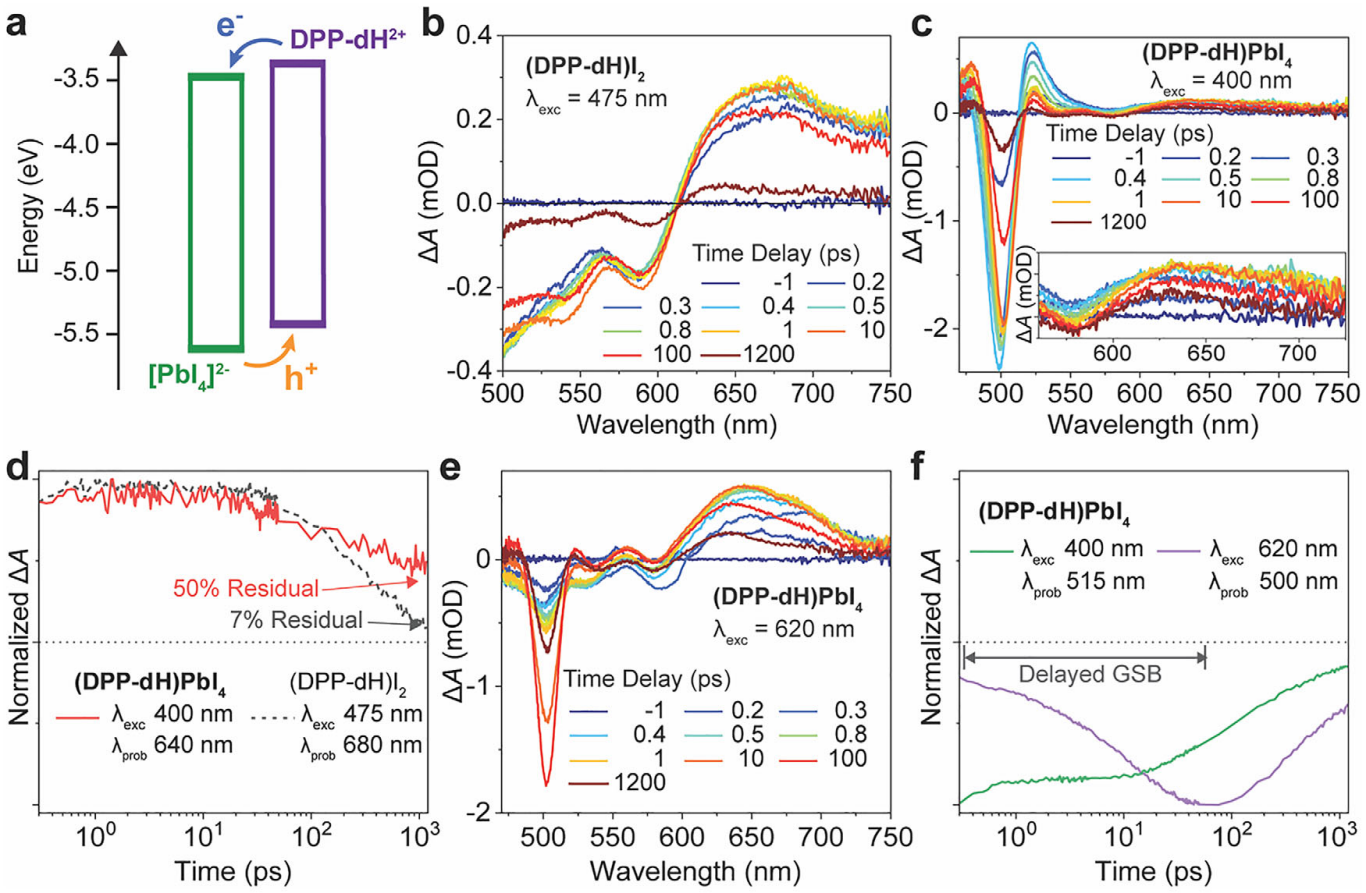
(a) Electron energy level diagram of the inorganic [PbI_4_]^2−^ and DPP‐dH^2+^ determined from UPS and absorption band‐edge analysis. Arrows indicate the complimentary electron/hole transfer pathways upon selective excitation. (b) Transient absorption spectra of (DPP‐dH)I_2_ films at cation excitation (*λ* = 475 nm). (c) Transient absorption spectra of **(DPP‐dH)PbI_4_** under predominantly perovskite excitation (*λ* = 400 nm). The enlarged photoinduced absorption (PA) region is shown in the inset. (d) Comparison of the PA dynamics of (DPP‐dH)I_2_ and **(DPP‐dH)PbI_4_** probed at the maximum of the PA band. (e) Transient absorption spectra of **(DPP‐dH)PbI_4_** under predominantly cation excitation (*λ* = 620 nm). (f) Comparison of the GSB dynamics of **(DPP‐dH)PbI_4_** under perovskite versus cation excitation.

To further validate the type‐II heterojunction structure, transient absorption spectroscopy (TAS) was first performed on the pure DPP‐dH spacer (iodide salt) film (Figure 2b). Upon excitation at 475 nm, characteristic features of a ground state bleach (GSB) from 500 to 610 nm and photoinduced absorption (PA) starting at 610 nm and extending into the NIR were observed. The shape of the GSB mirrors the absorption profile, and both the GSB and PA components exhibit a rapid sub‐nanosecond decay.

In contrast to the pure organic spacer, TAS on **(DPP‐dH)PbI_4_** films at *λ*_exc_ = 400 nm produces a sharp GSB at 500 nm (Figure 2c), which is consistent with the response of **(PEA)_2_PbI_4_** under identical excitation (Figure S4). This GSB is attributed to depopulation of the ground state of the inorganic [PbI_4_]^2−^ slab. In the **(DPP‐dH)PbI_4_** film, additional weak GSB (560 to 600 nm) and PA (600 to 750 nm) features are observed (Figure 2c, inset). We note that upon excitation at 400 nm, absorption is dominated by the inorganic perovskite slab but a minor contribution from direct excitation from the organic spacer is expected. Although the GSB of the [PbI_4_]^2−^ decays rapidly, similar to the behavior observed in **(PEA)_2_PbI_4_** (Figure S4b), the PA feature at 640 nm in the layered perovskite exhibits markedly prolonged kinetics compared to the pure spacer film. Approximately 50 % of the population persists after 1 ns, compared to 7 % in the pure spacer film, as shown in the kinetic traces of Δ*A* at the respective probe wavelengths (*λ*_prob_) in Figure 2d. As seen in **(PEA)_2_PbI_4_** (Figure S4), no significant PA is expected at 640 nm from the [PbI_4_]^2−^ slabs, so this is related to PA from the organic spacer. The prolonged lifetime relative to the neat spacer film strengthens the assignment of this PA to charge‐separated states generated via interfacial hole transfer from the inorganic [PbI_4_]^2−^ slab to the organic spacer, rather than direct photoexcitation of the organic component, supporting the view that effective charge separation and stabilization of the free carriers is occurring in the hybrid structure.

Conversely, when **(DPP‐dH)PbI_4_** films are excited at 620 nm, where absorption by [PbI_4_]^2−^ slabs is negligible (see Figure S5), a perovskite GSB is still observed (Figure 2e). In contrast to the faster risetime observed upon 400 nm excitation, the perovskite GSB exhibits a slower rise, reaching its maximimum only at ca. 50 ps (see kinetic trace Figure 2f). This delayed onset provides strong evidence for electron transfer from the excited organic spacer to the [PbI_4_]^2−^ slab. Furthermore, a long‐lived PA signature from the spacer is confirmed, with 40 % persisting beyond the experimental timescale, further suggesting the occurance of electron transfer phenomena. Possible alternative mechanisms are also discussed in the Supplementary Note (Supporting Information), although the available experimental evidence is most consistent with the bidirectional charge‐transfer mechanism. Overall, the TAS data demonstrate efficient bidirectional charge separation, regardless of whether excitation selectively targets the inorganic perovskite or the organic spacer.

Encouraged by the TAS results supporting bidirectional photoexcited charge transfer of **(DPP‐dH)PbI_4_**, we conducted flash‐photolysis time‐resolved microwave conductivity (fp‐TRMC) measurements to investigate the photogenerated carrier properties. Monitoring the decay of thin film photoconductance upon 500 nm excitation, as shown in Figure 3a, reveals a fast mono‐exponential decay with a time constant of 35 ns for **(PEA)_2_PbI_4_**. In contrast, **(DPP‐dH)PbI_4_** exhibits a remarkably prolonged bi‐exponential decay with time constants of 230 ns and 1.8 µs. In both **(PEA)_2_PbI_4_** and **(DPP‐dH)PbI_4_**_,_ the fast, ns time constant can be attributed to exciton relaxation in the inorganic slabs [19]. The extended photoconductivity lifetime in the case of **(DPP‐dH)PbI_4_** consists of charge separation resulting from the type‐II heterojunction [51, 52]. Importantly, similar biexponential decay with prolonged charge carrier lifetime is observed for **(DPP‐dH)PbI_4_** excited into the organic spacer at 600 nm (Figure S6), supporting the bidirectional transfer of holes and electrons upon selective excitation of either component.

**FIGURE 3:**
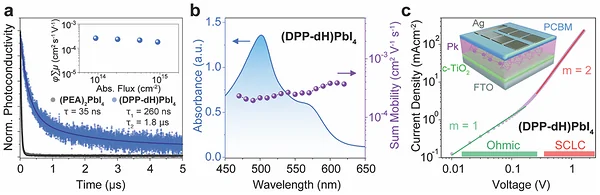
(a) The fp‐TRMC transient decays of **(DPP‐dH)PbI_4_** and **(PEA)_2_PbI_4_** at an absorbed photon flux of 10^14^ cm^−2^ (*λ*_exc_ = 500 nm). The obtained mobilities of **(DPP‐dH)PbI_4_** with different photon flux is shown in inset. (b) The photoexcited mobilities of **(DPP‐dH)PbI_4_** with different incident wavelengths. The nearly invariant peak photoconductivity across excitation wavelengths indicates that the carrier mobilities are not limited by variations in film absorbance. (c) The electron‐only SCLC *J*‐*V* curve of **(DPP‐dH)PbI_4_** with the corresponding device architecture used for the measurement shown in the inset.

The product of the free charge carrier generation quantum yield (*φ*) and the sum of the mobilities (Σ*μ*), *φ*Σ*μ*, was estimated from the peak photoconductivity (see experimental section, Supporting information). For **(DPP‐dH)PbI_4_**_,_ *φ*Σ*μ* reaches 2.6 × 10^−4^ cm^2^V^−1^s^−1^ at an absorbed photon flux of 10^14^ cm^−2^ (Figure 3a, inset). The sum mobility follows the expected behavior of increasing magnitude with decreasing excitation photon flux, attributable to reduced exciton–exciton annihilation, eventually reaching a plateau in the log–log plot. Significantly, the *φ*Σ*μ* values remain similar when the excitation wavelength is varied between 475 and 620 nm (Figure 3b). This wavelength independence provides strong supporting evidence for the complementary charge separation confirmed by TAS.

We note that the measured *φ*Σ*μ* for **(DPP‐dH)PbI_4_** is one order of magnitude lower than that of **(PEA)_2_PbI_4_** (Figure S7). However, we note that our fp‐TRMC measurement probes mobility parallel to the substrate. Since the layers are preferentially aligned with the substrate, the presence of photogenerated holes in the organic spacer layer of **(DPP‐dH)PbI_4_** could lead to a limitation in electronic communication between adjacent DPP molecules. Thus, compared to **(PEA)_2_PbI_4_** where, given the type‐I nano‐heterojunction electronic structure, photogenerated charges are confined and delocalized exclusively within the inorganic layers [34], a lower sum mobility in **(DPP‐dH)PbI_4_** is expected. However, the long carrier lifetimes and the possibility of the organic layer to transport charge associated with the type‐II nano‐heterojunction architecture suggest that **(DPP‐dH)PbI_4_** should exhibit enhanced charge separation and improved out‐of‐plane electron conductivity. To evaluate this, we fabricated electron‐only space‐charge‐limited current (SCLC) devices in which **(DPP‐dH)PbI_4_** or **(PEA)_2_PbI_4_** were sandwiched between *c*‐TiO_2_ (compact‐titanium dioxide) and PCBM ([6, 6]‐phenyl‐C61‐butyric acid methyl ester), as illustrated in Figure 3c. Similar to NDI‐based layered perovskites [32], the **(DPP‐dH)PbI_4_** devices display no trap‐filled limit (TFL) region following the initial ohmic response. Using the maximum‐trap‐density relation (see experimental section), the maximum trap density in **(DPP‐dH)PbI_4_** is estimated to be 3.3 × 10^14^ cm^−3^. In contrast, a pronounced TFL region is observed for **(PEA)_2_PbI_4_**, corresponding to a trap density of 6.8 × 10^15^ cm^−3^ (Figure S8), which is similar to previously reported value [53]. This result indicates a substantially smaller trap density in **(DPP)PbI_4_** than **(PEA)_2_PbI_4_** and other small‐molecule‐based layered perovskites [54].

The electron mobility, *µ*_e_, was extracted from SCLC data using the Mott–Gurney law, with the film thickness determined from cross‐sectional SEM (Figure S9) and device capacitance obtained by impedance spectroscopy (Figure S10). Averaging across multiple devices (Figure S11), **(DPP‐dH)PbI_4_** exhibits an out‐of‐plane electron mobility of (1.17 ± 0.3) × 10^−3^ cm^2^ V^−1^ s^−1^, with champion values reaching 1.3 × 10^−3^ cm^2^.V^−1^ s^−1^, comparable to the value reported for NDI‐based lead layered perovskite [55]. To confirm these values, a pulsed‐voltage‐sweep SCLC measurements were also performed. Using 50 ms voltage pulses with dwell times ranging from 0.1 to 60 s, no significant changes in the *J*–*V* characteristics were observed (Figure S12), confirming that the extracted *µ*_e_ of **(DPP‐dH)PbI_4_** was not affected by ion migration [56]. Notably, this value is approximately one order of magnitude higher than that of **(PEA)_2_PbI_4_**, (*µ*_e_ = (4.40 ± 1.2) × 10^−4^ cm^2^ V^−1^ s^−1^), consistent with previously reported values [57, 58].

In addition to the improved optoelectronic properties, **(DPP‐dH)PbI_4_** also exhibits enhanced moisture resistance compared to the conventional **(PEA)_2_PbI_4_** analogue. Water contact‐angle measurements (Figure S13) show a significantly higher initial contact angle for **(DPP‐dH)PbI_4_** (87^°^) than for **(PEA)_2_PbI_4_** (58^°^), together with only a minor decrease after 1 min (87^°^ to 83^°^), whereas the contact angle of **(PEA)_2_PbI_4_** decreases substantially (58^°^ to 25^°^). Consistent with its greater hydrophobicity, **(DPP‐dH)PbI_4_** also demonstrates improved environmental stability under continued exposure directly to air at 60 ^°^C and 60 % relative humidity (Figure S14). Although the excitonic absorption of **(PEA)_2_PbI_4_** rapidly decreases and broadens after only one day, **(DPP‐dH)PbI_4_** exhibits only a slight reduction in intensity after 3 days, indicating enhanced resistance to temperature and moisture‐induced degradation.

Finally, taking advantage of the complementary light absorption of the DPP‐incorporated layered perovskite and its strong spectral overlap with the solar spectrum, we fabricated p‐i‐n solar cells with a device architecture shown in Figure 4a (see energy diagram in Figure S15). Both the short‐circuit current (*J*_SC_) and open‐circuit voltage (*V*_OC_) of **(DPP‐dH)PbI_4_** are enhanced, with an average value of 0.67 mA cm^−2^ and 0.60 V, respectively, compared to 0.42 mA cm^−2^ and 0.29 V for **(PEA)_2_PbI_4_** under AM1.5G 1 sun illumination (100 mW cm^−2^). The **(DPP‐dH)PbI_4_** devices also exhibit a higher fill factor (FF) of 55.7 %, relative to 28.4 % for **(PEA)_2_PbI_4_** devices (Figure 4b, Table S1). The enhanced *J*_SC_ can be attributed to the extended visible absorption of **(DPP‐dH)PbI_4_**, which increases photocurrent generation. In addition, the reduced trap density and prolonged carrier lifetimes likely suppress nonradiative recombination, contributing to the improved *V*_OC_ and FF. Benefiting from these improved performance parameters, **(DPP‐dH)PbI_4_** achieves a five‐fold increase in power conversion efficiency (PCE) over **(PEA)_2_PbI_4_** (average PCE: 0.04 %), with a champion device reaching 0.26 % as demonstrated in Figure 4b. Although the PCE remains modest and lower than that of state‐of‐the‐art quasi‐2D perovskites, it is comparable to values typically reported for pure phase (*n* = 1) layered perovskites [59, 60].

**FIGURE 4:**
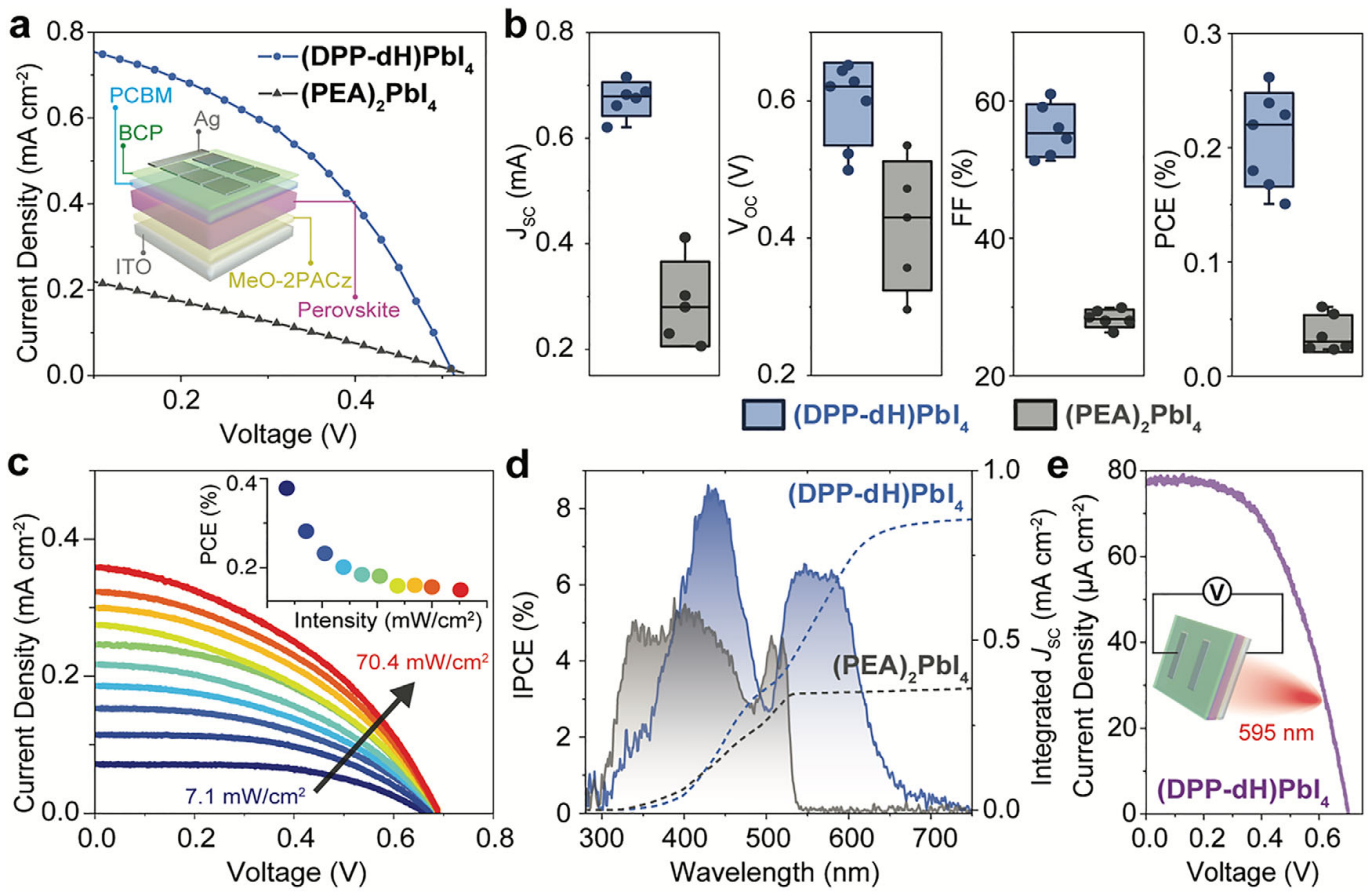
(a) J‐V curves of **(DPP‐dH)PbI_4_** and **(PEA)_2_PbI_4_** devices, the inset shows the p‐i‐n device architecture. (b) Statistical distributions of *J*_SC_, *V*_OC_, FF and PCE for **(DPP‐dH)PbI_4_** and **(PEA)_2_PbI_4_** devices. (c) *J*‐*V* curves of **(DPP‐dH)PbI_4_** measured under different illumination intensities, with the corresponding PCE shown in the inset. (d) The IPCE curves and the corresponding integrated *J*_SC_ curves of **(DPP‐dH)PbI_4_** and **(PEA)_2_PbI_4_**. (e) The *J*‐*V* curves of **(DPP‐dH)PbI_4_** under illumination at 595 nm, demonstrating extended visible light photon‐to‐current conversion.

Furthermore, varying the light intensity reveals an intriguing behavior in **(DPP‐dH)PbI_4_** devices, where the power conversion efficiency increases from 0.15 % to 0.38 % as the intensity of the simulated solar light is reduced from 70.4 to 7.1 mW cm^−2^ (Figures 4c, S16). This enhancement originates primarily from an increase in the fill factor at lower illumination intensities (Figure S17), which may be attributed to reduced resistive losses with the limited out‐of‐plane conductivity of *n* = 1 layered perovskites [61]. Indeed, extracting the shunt resistance, *R*_sh_, from the *J‐V* curves in Figure 4c reveals a significant decrease in *R*_sh_ with increasing light intensity (Figure S18). This is consistent with the view that under high illumination intensities, the limited out‐of‐plane transport leads to carrier accumulation and increased transport through defect‐mediated leakage pathways, such as grain boundaries and interfaces. Nevertheless, such improved performance under low light intensity suggests that **(DPP‐dH)PbI_4_** may eventually be well suited for indoor photovoltaic applications.

To verify that light absorption by the organic spacer contributes to photocurrent generation and to demonstrate the extended spectral response by **(DPP‐dH)PbI_4_**, incident photon‐to‐current efficiency (IPCE) spectra were measured. As shown in Figure 4d, the IPCE of **(PEA)_2_PbI_4_** sharply decreases beyond *λ* ≥ 520 nm, whereas the **(DPP‐dH)PbI_4_** device exhibits measurable IPCE values up to 650 nm, reflecting the expanded absorption window introduced by the DPP spacer. This behavior is consistent with the TRMC measurements, which show that photoexcited carrier mobility persists at excitation wavelengths up to 650 nm.

Integration of the IPCE spectra with the AM1.5G spectrum yields predicted *J*_SC_ very close to the values measured under one sun illumination, confirming the values of PCE reported. Deconvolution of the IPCE spectrum indicates that approximately 44 % of the integrated photocurrent originates from the DPP‐dH^2+^ absorption region (Figure S19), highlighting the substantial contribution of the organic spacer to photocurrent generation. However, the relatively low absorbed photon‐to‐current efficiency (APCE, ∼15 %) as shown in Figure S20 indicates that a substantial fraction of absorbed photons does not contribute to collected photocurrent, further suggesting that the modest PCE of **(DPP‐dH)PbI_4_** could be substantially improved (e.g., by affording a preferential vertical orientation of the layered perovskite).

We note that a pronounced dip in the IPCE of **(DPP‐dH)PbI_4_** is observed around 510 nm, which corresponds to the inorganic layer excitonic absorption peak (see overlayer of IPCE and absorption in Figure S21). Similar dip features are consistently observed across multiple devices for both **(DPP‐dH)PbI_4_** and **(PEA)_2_PbI_4_** (Figure S22), indicating that the excitonic transitions in these layered perovskites are optically active but contribute less effectively to photocurrent generation. We attribute this behavior to the formation of strongly bound, Frenkel‐like excitons in the inorganic layers at the excitonic absorption maximum. This behavior is consistent with the intrinsically large exciton binding energies of *n* = 1 layered perovskites arising from quantum and dielectric confinement [62]. The presumed high binding energy of these excitons can limit their dissociation into free carriers, therefore contributing less efficiently to the photocurrent [63], and consequently, a dip in the IPCE. A similar phenomenon has also been reported in type‐II organic‐inorganic quantum dot systems, where charge‐transfer excitons can instead promote electron‐hole recombination rather than efficient charge separation [64]. Comparing **(DPP‐dH)PbI_4_** and **(PEA)_2_PbI_4_**, although **(DPP‐dH)PbI_4_** exhibits a higher dielectric constant than **(PEA)_2_PbI_4_**, its larger interlayer spacing may enhance exciton confinement [65], leading to stronger exciton binding and consequently a more pronounced IPCE dip in the case of **(DPP‐dH)PbI_4_**. Future work employing temperature‐resolved spectroscopy or spacer dielectric engineering would be needed to fully understand this aspect and to develop a strategy to convert these otherwise photocurrent‐inactive excitons into extractable carriers.

Finally, to directly demonstrate the contribution of the extended light absorption in **(DPP‐dH)PbI_4_**, *J‐V* measurements under monochromatic 595 nm illumination were performed with 31.4 mW cm^−2^ flux intensity. Although **(PEA)_2_PbI_4_** exhibits no detectable photocurrent at this excitation wavelength, **(DPP‐dH)PbI_4_** generates a clear photocurrent response (Figure 4e), with a *J*_SC_ and *V*_OC_ of 79.1 µA/cm^2^ and 0.7 V, respectively, with a PCE of 0.09 %. Together, these results highlight the strong potential of DPP‐based visible‐light‐absorbing spacers to enable layered perovskites with broadened spectral utilization for next‐generation optoelectronic applications.

## 3 Conclusion

In this work, we demonstrate that incorporating a visible‐light‐absorbing π‐conjugated dithiophene‐DPP spacer with a length‐optimized dihexyl ammonium linker enables the formation of a type‐II layered perovskite **(DPP‐dH)PbI_4_**. Supported by comprehensive optical and electrical measurements, **(DPP‐dH)PbI_4_** exhibits complementary charge transfer under selective excitation of either the inorganic slabs or the organic spacer, leading to long‐lived free carriers, reduced trap density, and enhanced electron mobility compared to that of **(PEA)_2_PbI_4_**. Importantly, the extended visible absorption of **(DPP‐dH)PbI_4_** directly translates into improved photovoltaic performance and an expanded photo response up to 650 nm (1.9 eV), addressing a key limitation of conventional *n* = 1 layered perovskites. These findings establish visible‐light‐active semiconducting spacers as an effective strategy to improve performance and provide valuable design principles for advancing layered hybrid organic–inorganic perovskites toward next‐generation optoelectronic applications.

## Conflicts of Interest

The authors declare no conflicts of interest.

## Supporting information




**Supporting File**: adma74681‐sup‐0001‐SuppMat.pdf.

## Data Availability

Data for this article, including data tables used to create figures in the main text, are available on Zenodo at https://doi.org/10.5281/zenodo.20268369.
