# Supplementary material for: Diketopyrrolopyrrole‐Based Cation as a Semiconducting Spacer in Layered Perovskite
Source: Adv Mater. 2026 Aug 18;38(58):e74681. doi: 10.1002/adma.74681 (PMC13674623; doi:10.1002/adma.74681)
Supplement: Supplementary file 1 [file ADMA-38-e74681-s001.pdf]

Supporting Information for:

Diketopyrrolopyrrole-Based Cation as a Semiconducting Spacer in Layered Perovskite

Waygen Thor,[1‖] Colin Jeanguenat,[1‖] Louise De Cian,[1] Demetra Tsokkou,[2] Parnian Ferdowsi,[1] Nicolas J. Diercks,[1] Natalie Banerji,[2] Jun-Ho Yum,[1*] and Kevin Sivula[1*]

[1] Laboratory for Molecular Engineering of Optoelectronic Nanomaterials (LIMNO), Institut des Sciences et Ingénierie Chimiques (ISIC), École Polytechnique Fédérale de Lausanne (EPFL), 1015 Lausanne, Switzerland.

[2] FemtoMat Research Group, Department für Chemie, Biochemie und Pharmazie, University of Bern, Freiestrasse 3, 3012 Bern, Switzerland.

‖ Contributed equally to this work.

Table of Contents

	Contents		Page
**1**	**Materials and Synthesis**		S2
	**Scheme S1.**	Synthesis of DPP with different chain length	S2
**2**	**Experimental**		S3
**3**	**Characterization**		S4
	**Figure S1.**	XRD and absorption of incorporation of DPP of different alkyl chain lengths with **PbI_2**.	S7
	**Figure S2.**	UPS and absorption edge analysis of **(DPP-dH)PbI_4** and (DPP-dH)I_2.	S7
	**Figure S3.**	Emission of **(DPP-dH)PbI_4**, (DPP-dH)I_2 and **$(PEA)_2PbI_4$**.	S8
	**Figure S4.**	Transient absorption spectra of **$(PEA)_2PbI_4$**.	S8
	**Figure S5.**	Absorption spectra of of **(DPP-dH)PbI_4** and (DPP-dH)I_2.	S8
	**Figure S6.**	Fp-TRMC decays of **(DPP-dH)PbI_4** with λ_{exc} = 600 nm.	S9
	**Figure S7.**	Peak sum mobilities of **(DPP-dH)PbI_4** and **$(PEA)_2PbI_4$**.	S9
	**Figure S8.**	Electron-only SCLC J-V curve of **$(PEA)_2PbI_4$**.	S9
	**Figure S9.**	Cross-sectional SEM of **(DPP-dH)PbI_4** and **$(PEA)_2PbI_4$**.	S10
	**Figure S10.**	Impedance spectrum of **(DPP-dH)PbI_4**.	S10
	**Figure S11.**	Electron mobilities of **(DPP-dH)PbI_4** extracted from SCLC.	S11
	**Figure S12.**	Pulsed SCLC J-V curve of **(DPP-dH)PbI_4**.	S11
	**Figure S13.**	Contact angle of **(DPP-dH)PbI_4** and **$(PEA)_2PbI_4$**.	S11
	**Figure S14.**	Absorption spectra and photograph of **(DPP-dH)PbI_4** and **$(PEA)_2PbI_4$** after high temperature and humidity environment.	S12
	**Figure S15.**	Flat energy diagram of p-i-n solar cell device of **(DPP-dH)PbI_4**.	S12
	**Table S1.**	Average PCE, J_{SC}, V_{OC} and FF of **(DPP-dH)PbI_4** solar device.	S13
	**Figure S16.**	J-V curve and PCE of **(DPP-dH)PbI_4** solar device with different illuminating intensity.	S13
	**Figure S17.**	Evolution of J_{SC}, V_{OC} and FF of **(DPP-dH)PbI_4** solar device with different illuminating intensity.	S13
	**Figure S18.**	Evolution of shunt resistance with different illuminating intensity.	S14
	**Figure S19.**	IPCE curves of **(DPP-dH)PbI_4** and the corresponding integrated J_{SC} curves contributed from the inorganic slabs and DPP-dH^{2+} absorption.	S14
	**Figure S20.**	APCE curve of **(DPP-dH)PbI_4**.	S14

	**Figure S21.**	Comparison of absorption and IPCE spectra of **(DPP-dH)PbI$_4$** and **(PEA)$_2$PbI$_4$**.	S15
	**Figure S22.**	IPCE spectra of **(DPP-dH)PbI$_4$** and **(PEA)$_2$PbI$_4$** over repeated measurements.	S15
**4**	**Supplementary Note**		S15
**5**	**References**		S16
**6**	**NMR Spectra**		S17

1. Materials and Synthesis

3,6-Di(2-thienyl)-2,5-dihydropyrrolo[3,4-c]pyrrole-1,4-dione (DPP) was obtained from BLDPharm and NBoc-1,6-diaminohexane were obtained from Fluorochem. Perovskite precursor solutions were prepared with lead (II) iodine 99.99 % from Sigma Aldrich. All solvents and chemicals were used without purification.

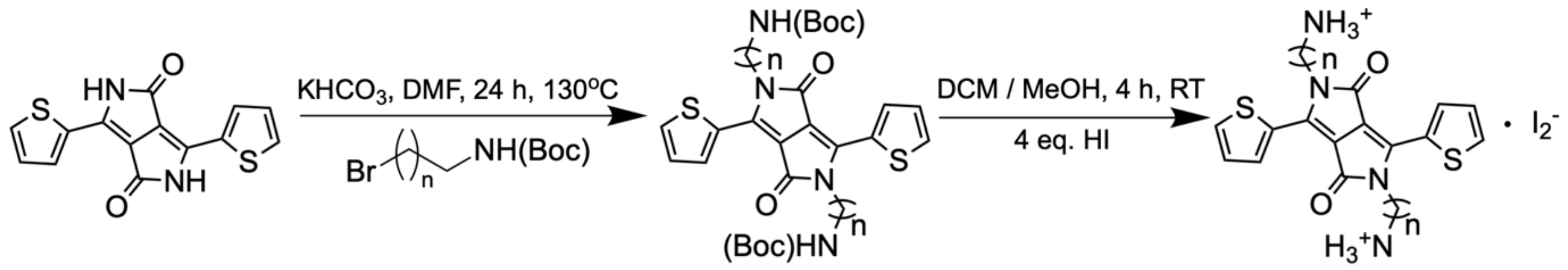

Scheme S1. The synthetic route of (DPP-dP)I$_2$ (n = 3), (DPP-dB)I$_2$ (n = 4) and (DPP-dH)I$_2$ (n = 6).

The reaction conditions were inspired by previous literature.[1,2] DPP (2.12 g, 6.71 mmol, 1 eq.) and potassium carbonate (2.94 g, 21.2 mmol, 3.16 eq.) were added in a two-neck flask. The reactor was put under argon and dry DMF (70.7 mL) was added. The mixture was stirred for 1h at 130°C before adding the Boc-protected amine halogenoalkane. For DPP-dP: tert-butyl 3-bromopropylcarbamate, for DPP-dB: 4-(Boc-amino)butyl bromide, and for DPP-dH: N-boc-6-bromo-hexylamine (17.7 mmol, 2.6 eq.).

The reaction was carried out for 24 h at 130 °C, and the crude was poured into water (276 mL). After stirring for 30 min at room temperature, DCM was used to extract the water. The combined organic phases were extracted with 10% by weight LiCl three times to remove the DMF traces. The organic phase was dried with MgSO$_4$. The crude solid was purified by chromatography (wet load with DCM, eluent: DCM to get rid of the fast-moving impurities, then DCM 8 : ethyl acetate 2 v). A second column was performed with DCM 9.5 v and acetone 0.5 v with no pressure applied to yield the N-Boc protected DPP spacer in a yield of around 40% for all alkyls.

N-Boc protected DPP-dP.
^{1}H NMR (400 MHz, CDCl3) δ 8.94 (2H, d), 7.68 (2H, d), 7.32 (2H, dd), 5.29 (2H, broad), 4.20 (4H, t), 3.21 (4H, apparent q), 1.95 (4H, dd), 1.47 (18H, s).
^{13}C NMR (101 MHz, CDCl3) δ 161.76, 156.04, 140.16, 135.60, 131.18, 129.42, 128.82, 107.57, 79.15, 39.22, 37.38, 30.34, 28.48.
HRMS (APCI/QTOF) m/z: $[M + Na]^+$ Calcd for $C_{30}H_{38}N_4NaO_6S_2^+$ 637.2125; Found 637.2123.

N-Boc protected DPP-dB.

^{1}H NMR (400 MHz, CDCl3) δ 8.91 (2H, d), 7.65 (2H, d), 7.29 (2H, dd), 4.62 (2H, broad), 4.12 (4H, t), 3.23-3.14 (4H, m), 1.83-1.75 (4H, m), 1.66-1.59 (4H, m), 1.44 (18H, s).

^{13}C NMR (101 MHz, CDCl3) δ 161.40, 155.93, 139.99, 135.38, 130.87, 129.57, 128.75, 107.69, 79.18, 41.68, 40.15, 28.42, 27.35 (2 different C according to the HSQC).

HRMS (APCI/QTOF) m/z: $[M + Na]^+$ Calcd for $C_{32}H_{42}N_4NaO_6S_2^+$ 665.2438; Found 665.2434.

N-Boc protected DPP-dH.

^{1}H NMR (400 MHz, CDCl3) δ 8.93 (2H, d), 7.66 (2H, d), 7.30 (2H, dd), 4.54 (2H, s, broad), 4.09 (4H, t), 3.11 (4H, t), 1.82-1.723 (4H, m), 1.55-1.35 (30H, m).

^{13}C NMR (101 MHz, CDCl3) δ 161.37, 155.8, 140.01, 135.31, 130.75, 129.72, 128.65, 107.70, 79.05, 42.00, 40.51, 29.97, 29.85, 28.44, 26.48, 26.37.

HRMS (APCI/QTOF) m/z: $[M + Na]^+$ Calcd for $C_{36}H_{50}N_4NaO_6S_2^+$ 721.3064; Found 721.3057.

The N-Boc protected DPPs were then deprotected by mixing with 4 molar equivalent HI (57% in water) in DCM:methanol (1:1) to obtain the DPP iodide salts. The mixtures were stirred for 4 hours. The precipitates was collected by centrifugation and washed with diethyl ether and DCM until no reddish pink liquid could be removed for all alkyls.

(DPP-dP)I₂.

^{1}H NMR (400 MHz, MeOD) δ 8.88 (2H, d), 7.98 (2H, d), 7.39 (2H, dd), 4.27 (4H, t), 3.06 (4H, t), 2.16 (4H, tt).

^{13}C NMR (101 MHz, MeOD) δ 161.83, 140.26, 135.45, 132.24, 128.86, 128.46, 107.17, 38.43, 36.86, 27.73.

HRMS (ESI/QTOF) m/z: $[M + H]^+$ Calcd for $C_{20}H_{23}N_4O_2S_2^+$ 415.1257; Found 415.1266.

(DPP-dB)I_2.

^{1}H NMR (400 MHz, MeOD) δ 8.88 (2H, d), 7.97 (2H, d), 7.39 (2H, dd), 4.20 (4H, t), 3.01 (4H, t), 1.90-1.74 (8H, m).

^{13}C NMR (101 MHz, MeOD) δ 161.40, 140.16, 135.18, 131.93, 129.06, 128.29, 107.19, 40.76, 38.90, 26.55, 24.44.

(DPP-dH)I_2.

^{1}H NMR (600 MHz, MeOD) δ 8.84 (2H, d), 7.93 (2H, d), 7.35 (2H, dd), 4.08 (4H, t), 2.95 (4H, t), 1.76 (4H, m), 1.68 (4H, m), 1.51-1.44 (8H, m).

^{13}C NMR (151 MHz, MeOD) δ 161.22, 140.13, 135.11, 131.88, 129.23, 128.16, 107.08, 41.43, 39.27, 29.23, 27.04, 25.85, 25.55.

HRMS (ESI/QTOF) m/z: $[M + H]^+$ Calcd for $C_{26}H_{35}N_4O_2S_2^+$ 499.2196; Found 499.2191.

2. Experimental

Substrate cleaning. Both glass substrates and FTO substrates used were washed with soap-water (Hellmanex), deionized water, acetone, and isopropanol for 20 mins by ultrasound sequentially. The substrates were processed with UV-ozone for 15 mins before being used.

Thin film preparation. The precursor solution of **(DPP-dP)PbI_4**, **(DPP-dB)PbI_4**, and **(DPP-dH)PbI_4** film was prepared by mixing 0.2 M PbI_2 and 0.2 M of the respective cations in an anhydrous solution of DMF. The solution was stirred at 70 °C overnight to ensure homogeneity. The films were prepared by spin coating the solution at 2000 rpm (9 s ramping) for 30 s, followed by 4000 rpm (2 s ramping) for 30 s with chlorobenzene added at 17s before the program ends. The films were annealed at 160 °C for 1 hour under N_2 conditions.

For **$(PEA)_2PbI_4$**, the precursor solution was prepared by using the same concentration and solution but with 0.2 M PbI_2 and 0.4 M of PEAI stoichiometry. The films were prepared according to previous literature[3] and were annealed at 80 °C.

Device Fabrication. The electron-only devices were fabricated on a FTO/*c*-TiO_2/Perovskite/[6,6]-phenyl-C61-butyric acid methyl ester (PCBM)/Ag architecture as shown in Figure 3. The *c*-TiO_2 was deposited by spray-pyrolysis and annealed according to the literature.[4] The perovskite layer was deposited as described above. The PCBM electron transport layer was coated according to previous report,[5] with 10 mg of PCBM dissolved in 1 mL of chlorobenzene and spun-coated at 1000 rpm (1 s ramping) for 30 s and subsequently annealed at 100 °C for 10 mins. The 100 nm thick Ag electrode was thermally deposited via the controllable power.

The solar cells were fabricated on a FTO/2-(3,6-dimethoxy-9H-carbazol-9-yl)ethyl)phosphonic acid (MeO-2PACZ)/Perovskite/PCBM/Bathocuproine (BCP)/Au architecture. The MeO-2PACZ monolayer was deposited according to literature[6] with 0.5mg/mL concentration in ethanol, spun-coated at 1000 rpm at 1 s and subsequently annealed at 100 °C for 10 mins. The perovskite layer was deposited as described above. The PCBM electron transport layer was coated according to previous report,[5] with 10 mg of PCBM dissolved in 1 mL of chlorobenzene and spun-coated at 1000 rpm (1 s ramping) for 30 s and subsequently annealed at 100 °C for 10 mins. The BCP layer (2mg/mL in ethanol) was deposited using dynamic spin coating at 4000 rpm. The 80 nm thick Au electrode was thermally deposited via the controllable power.

3. Characterization.

NMR Spectra. NMR Spectra were acquired at room temperature using a Bruker 400-MHz spectrometer with $CDCl_3$ or *d*-DMSO as the solvent and tetramethylsilane (TMS) as an internal standard.

X-ray diffraction. Thin film X-ray diffraction (XRD) measurements were taken in the Bragg−Brentano geometry using nonmonochromatic Cu Kα1 radiation on a Bruker D8 Vario instrument equipped with a LynxEYE XE detector. GIWAXS measurements conducted under ambient conditions using a D8 Discover Plus TXS with an Eiger 2D detector. An X-ray source (1.5406 Å) at an incidence angle of 2° was employed.

Ultraviolet Photoelectron Spectroscopy. Ultraviolet photoelectron spectroscopy (UPS) measurements were carried out on an Axis Supra (Kratos Analytical) using the monochromated Kα X-ray line of an aluminum anode. The pass energy was set to 20 eV with a step size of 0.1 eV. The samples were electrically grounded to limit charging effects.

Physical characterization. The UV-visible light absorption measurements were carried out with a PerkinElmer LAMDA 365 UV-Vis spectrophotometer. The emission spectra were measured on an Edinburg Instrument FLS1000 spectrometer equipped with a cooled PMT detector. The topology and the cross-sectional images were obtained using scanning electron microscopy (SEM) on Zeiss Merlin with Gemini II column In-Lens detectors. Contact angle measurements were performed at room temperature by using a Krüss drop shape analyzer (DSA25S) and analyzed by Krüss Advance software through the sessile drop method. The water droplet volume was kept at 2μL.

Transient Absorption. Femtosecond transient absorption measurements were performed using a Ti:Sapphire amplifier system (Astrella, Coherent) with output pulses of 35 fs duration, 800 nm wavelengths and 1 kHz repetition rate. For the TA measurements the beam is split into two components, the pump and the probe. To convert the wavelength of the pump beam to 475 nm and 620 nm an optical parametric amplifier (Opera, Coherent) was used. Pump pulses at 400 nm were also used, and generated via double frequency generation of the fundamental pulses in a BiBo crystal. The broad spectrum of the probe beam is achieved by strongly focusing the fundamental light into a sapphire crystal to generate white light. The optical path of the probe pulses compared to the one of the pump pulses is adjusted with a computer-controlled micrometer precision translation stage. Probe beam is split into a reference beam and a signal beam. Pump and signal probe beams are spatially and temporally overlapped on the sample position, where the size of the pump beam was 2-4 times bigger than the probe beam size to ensure that we probe uniform photoexcited species distribution. The transmitted probe pulses are directed and spectrally dispersed in a home-built prism spectrometer (Entwicklungsbüro Stresing, Berlin) and detected with back-thinned Silicon CCD camera (Hamamatsu S07030-0906) to probe the visible wavelengths from 480 nm to 750 nm. The pump pulses were chopped at 500 Hz to measure of transmission change of the probe pulses and increase the signal-to-noise ratio. The measurements were performed for a probe pulses polarization at magic angle with respect to one of the pump pulses to avoid any polarization effects.

Time-resolved microwave conductivity. Time-resolved microwave conductivity (TRMC) measurements were performed under resonant microwave conditions with a copper cavity similar to the one used in previous studies.[7] The samples were photoexcited by a 3-5 ns pulse of a wavelength-tunable optical parametric oscillator (OPO) coupled to a diode-pumped Q-switched Nd:YAG laser at a wavelength of 500 nm (50 Hz). The microwaves (X-band region, 8.4-8.7 GHz) were generated by a voltage-controlled oscillator. The change in microwave power ($\Delta P/P$) reflected by the cavity upon sample excitation was monitored by a Schottky Zero Bias detector and amplified with a low-noise voltage amplifier. $\Delta P/P$ can be correlated to the photoinduced change in the conductance of the sample, ΔG,

$$\Delta G = -\frac{1\Delta P}{KP} \quad \text{(S1)}$$

where K is the sensitivity factor derived from the frequency response of the resonant cavity using an analytical impedance model that considered both the cavity geometry and the substrate properties.[8] A K-factor of 62000 was estimated for all the measurements conducted herein.

The assessment of the peak sum mobility ($\varphi \sum \mu$) was then obtained by employing the formula,

$$\varphi \sum \mu = \frac{\Delta G}{\beta e I_0 F_A} \quad \text{(S2)}$$

where ΔG is the fractional change in reflected microwave power, β is the ratio of the inner microwave cavity dimensions (2.26), e is the elementary charge, and $I_0 F_A$ is the number of absorbed photons.

Space Charge-Limited Current Measurement. The space charge-limited current (SCLC) measurement was measured in forward direction from -0.1 V to 1.3 V using a source meter (Keithley 4200), with a step voltage of 10 mV and a delay time of 0.1 s. The active area is 0.16 cm^2. The minimal trap density, n_{min} can be estimated by[9,10]

$$n_{\text{min trap}} = 4\pi^2 \frac{k_B T}{q^2} \frac{\varepsilon_0 \varepsilon_r}{L^2} \quad \text{(S3)}$$

where k_B is the Boltzmann constant, T is the temperature, q is the elementary charge, ε_0 is the vacuum permittivity, ε_r is the relative permittivity, L is the thickness of the thickness of the film. The trap density of device with measurable trap-filled-limit voltage (V_{TFL}) can be calculated by

$$n_{\text{trap}} = \frac{2V_{\text{TFL}} \varepsilon_0 \varepsilon_r}{qL^2} \quad \text{(S4)}$$

where V_{TFL} is the trap-filled-limit voltage.

The electron mobility can be estimated from the Mott-Gurney law,

$$J = \frac{9}{8} \varepsilon_0 \varepsilon_r \mu \frac{V^2}{L^3} \quad \text{(S5)}$$

where J is the current density and V is the applied voltage.

Impedance Spectroscopy. Impedance spectroscopic data were obtained with a Bio-Logic SAS SP 200 potentiostat with a scanned frequency range from 4×10^6 Hz to 10 Hz.

Photovoltaic Measurement. Photovoltaic devices were measured in forward direction from −0.1 to 1.3 V using a source meter (Keithley 4200) under AM 1.5 illumination (100 mW cm^{-2}) from a Sol3A class AAA solar simulator (Oriel 69920), with a step voltage of 10 mV and a delay time of 0.1 s. The light source intensity was calibrated using a standard Si solar cell. The active area defined by a metal mask aperture is 0.04 cm^2. The incident photon-to-current efficiency (IPCE) spectra were measured using an Oriel IQE-EQE 200B. For fluence dependent J-V curve, the scanning was from -0.1 to 1.3 V using a Bio-Logic SAS SP 50 potentiostat with Pico LED solar simulator (G2V Optics). For the illumination under 595 nm,

the scanning was from -0.1 to 1.3 V using a Bio-Logic SAS SP 50 potentiostat with single wavelength LED of 595 nm (Thorlabs) with illumination power of 31.4 mW cm^{-2}.

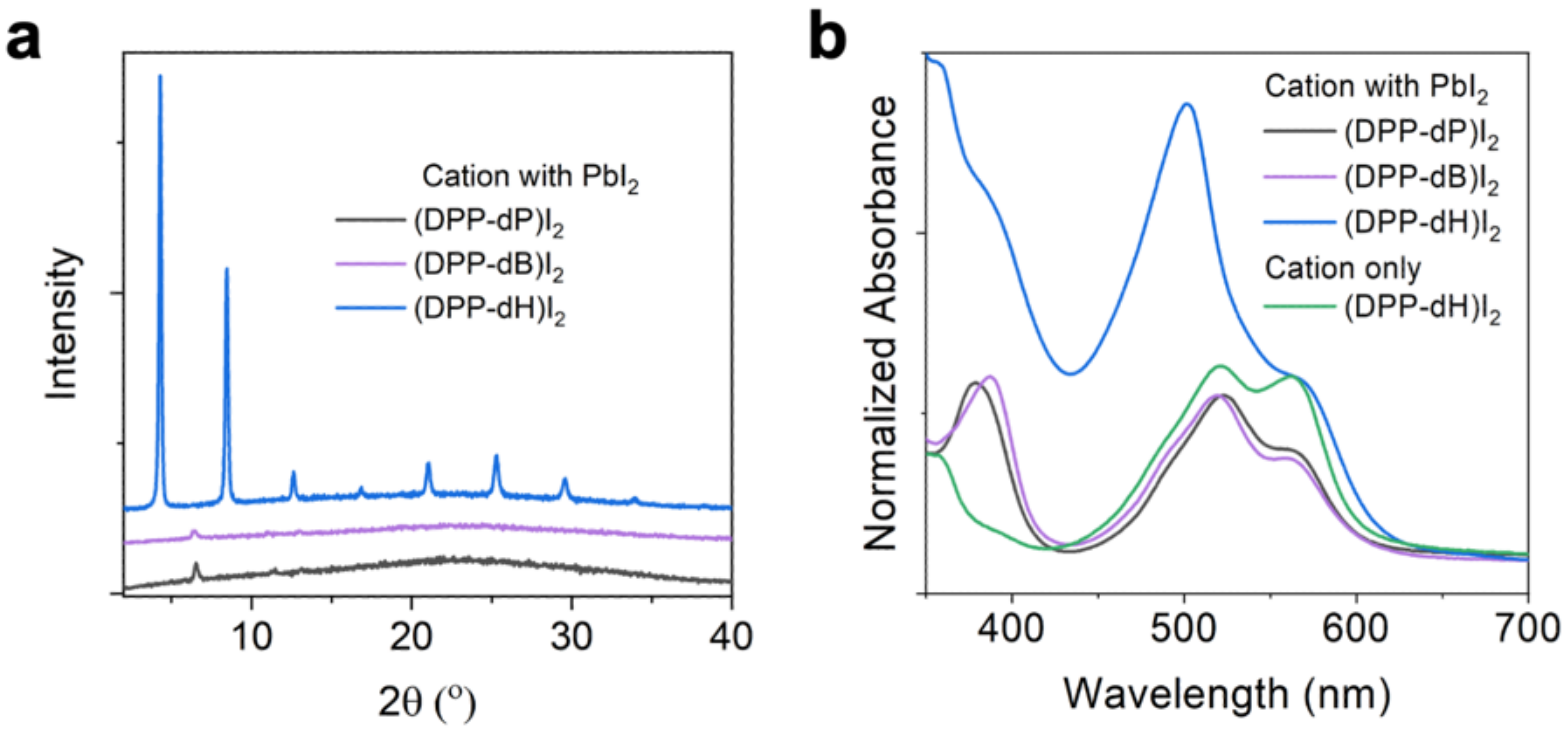

Figure S1. The (**a**) XRD and (**b**) absorption spectra of DPP cations with different alkyl lengths incorporated with PbI_2.

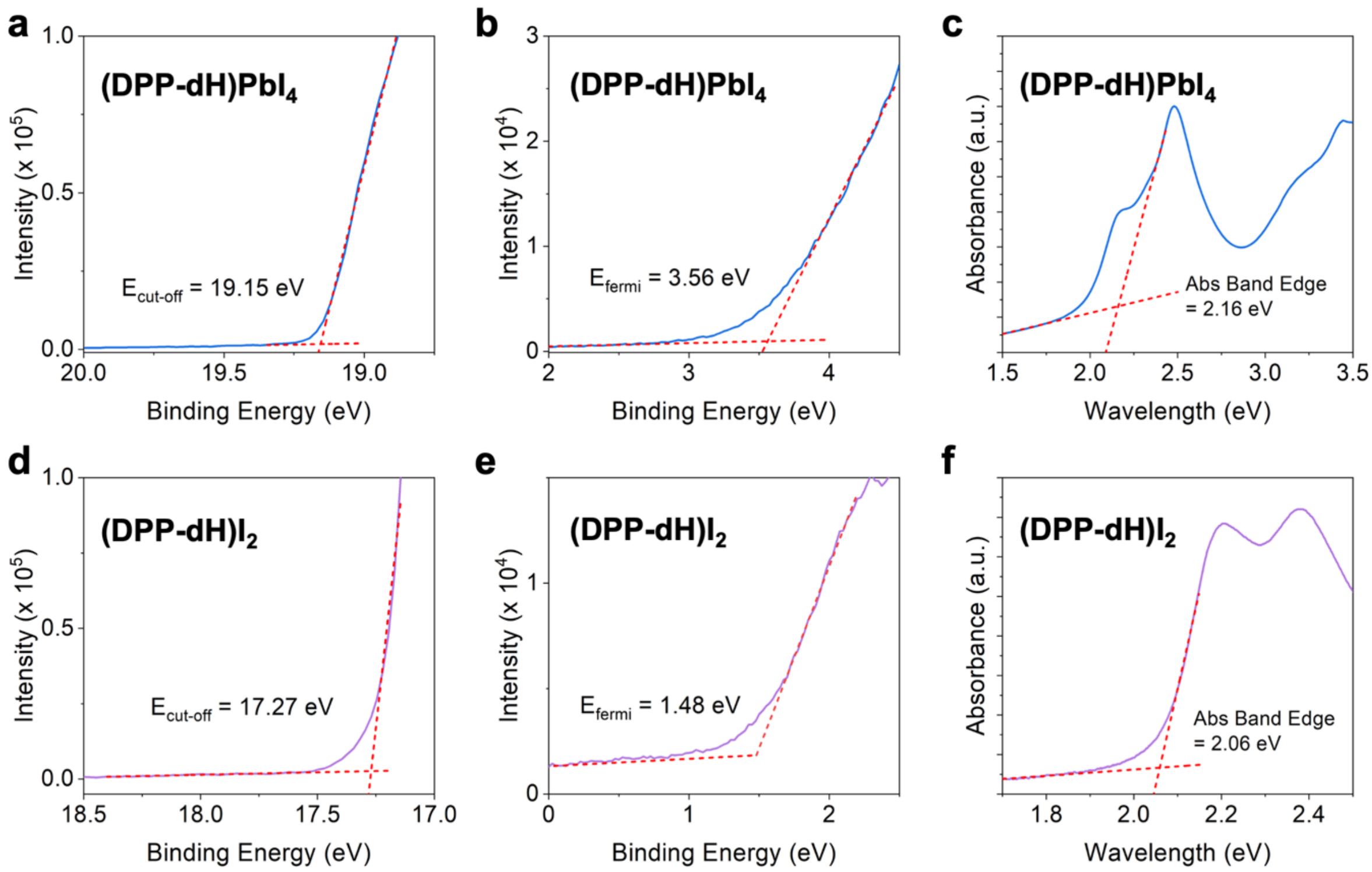

Figure S2. The UPS spectra showing the (**a**, **d**) secondary electron cut off and (**b**, **e**) valence band maximum region of **$(DPP\text{-}dH)PbI_4$** and $(DPP\text{-}dH)I_2$. The excitonic absorption band-edges are shown in (**c**, **f**), respectively.

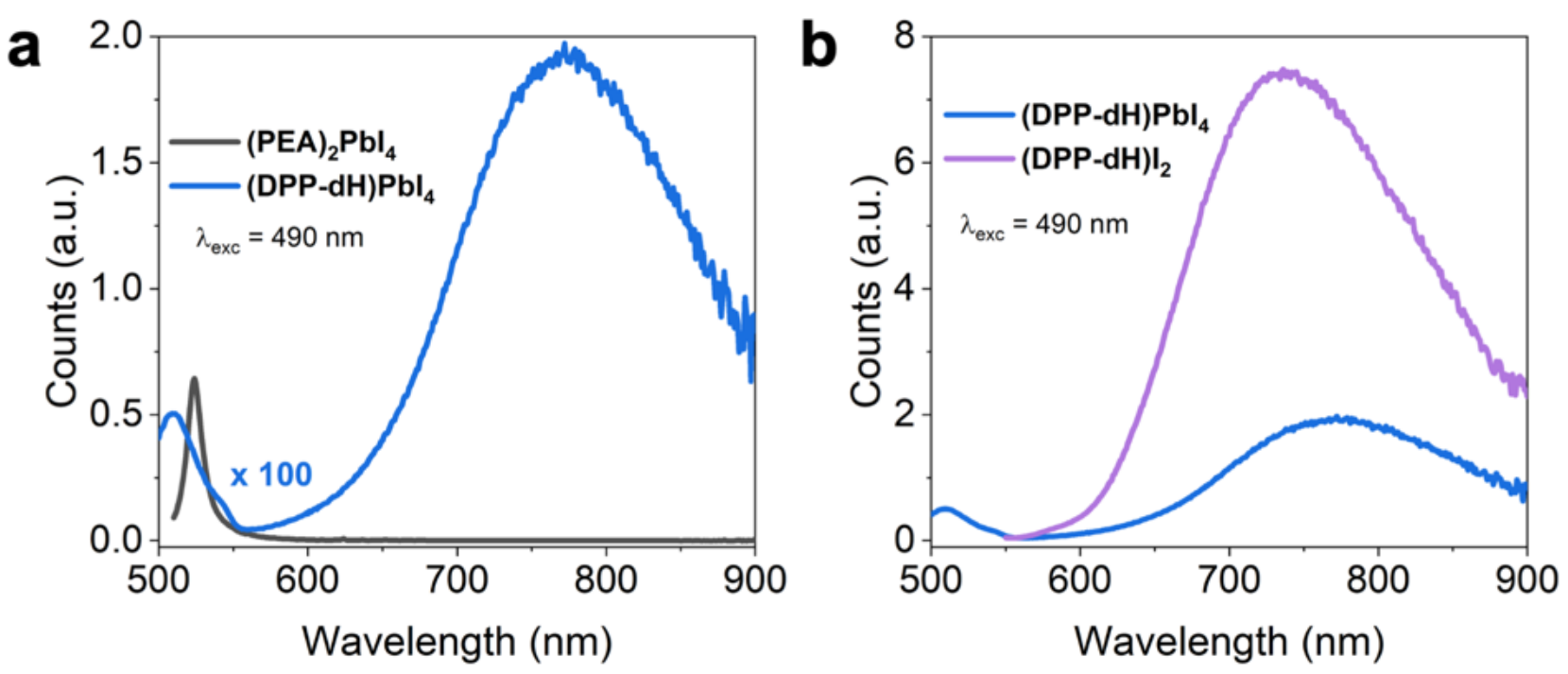

Figure S3. The emission of **(DPP-dH)PbI$_4$** compared to that of (**a**) **(PEA)$_2$PbI$_4$** and (**b**) the (DPP-dH)I$_2$ films. λ_{exc} = 490 nm.

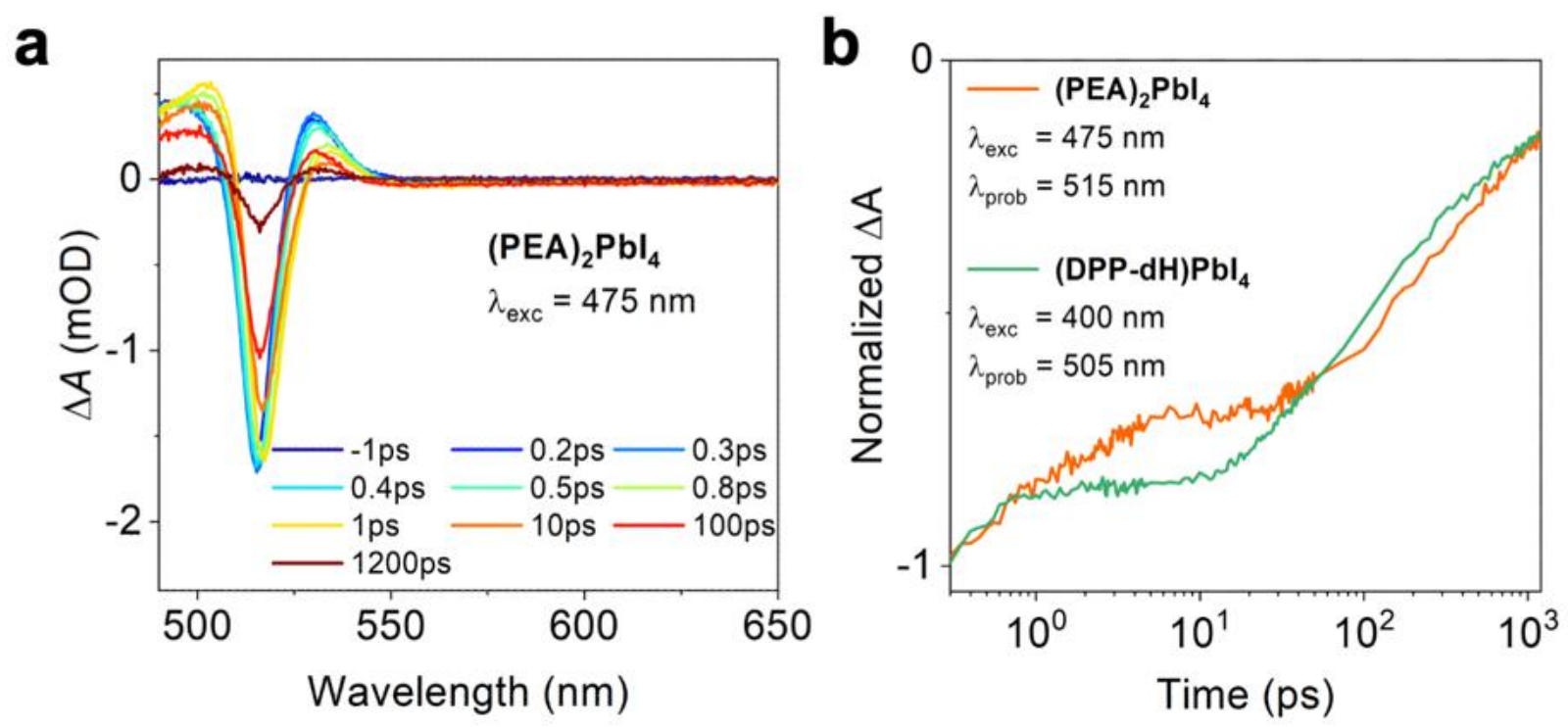

Figure S4. (**a**) Transient absorption spectra of **(PEA)$_2$PbI$_4$** under predominantly perovskite excitation (λ = 475 nm). (**b**) Comparison of the ground-state bleach (GSB) dynamics of **(PEA)$_2$PbI$_4$** and **(DPP-dH)PbI$_4$**.

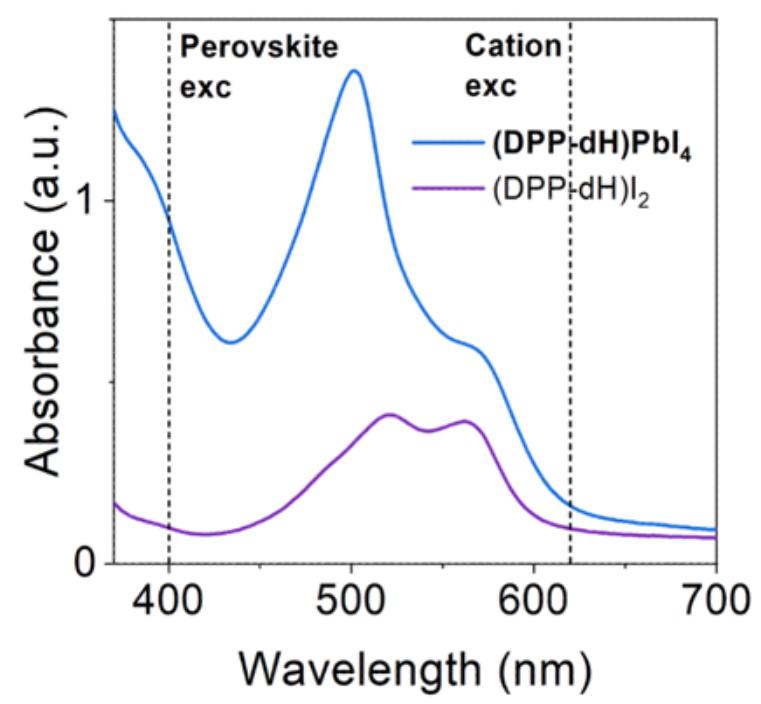

Figure S5. The absorption spectra of **(DPP-dH)PbI$_4$** and (DPP-dH)I$_2$ with the dashed line showing the pumping wavelength used in transient absorption spectroscopy for selective perovskite and cation excitation.

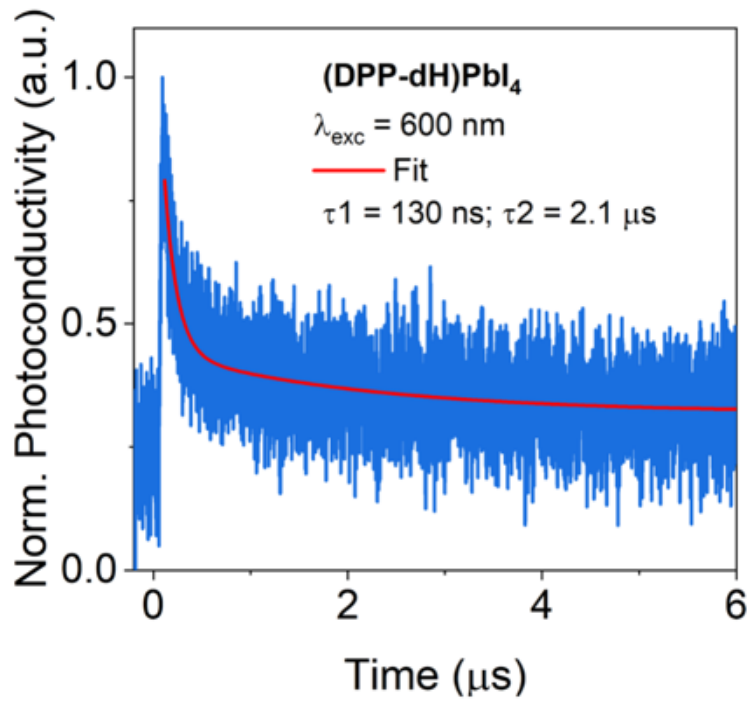

Figure S6. The fp-TRMC transient decays of **(DPP-dH)PbI4** at an absorbed photon flux of 10^{13} cm^{-2} (λ_{exc} = 600 nm).

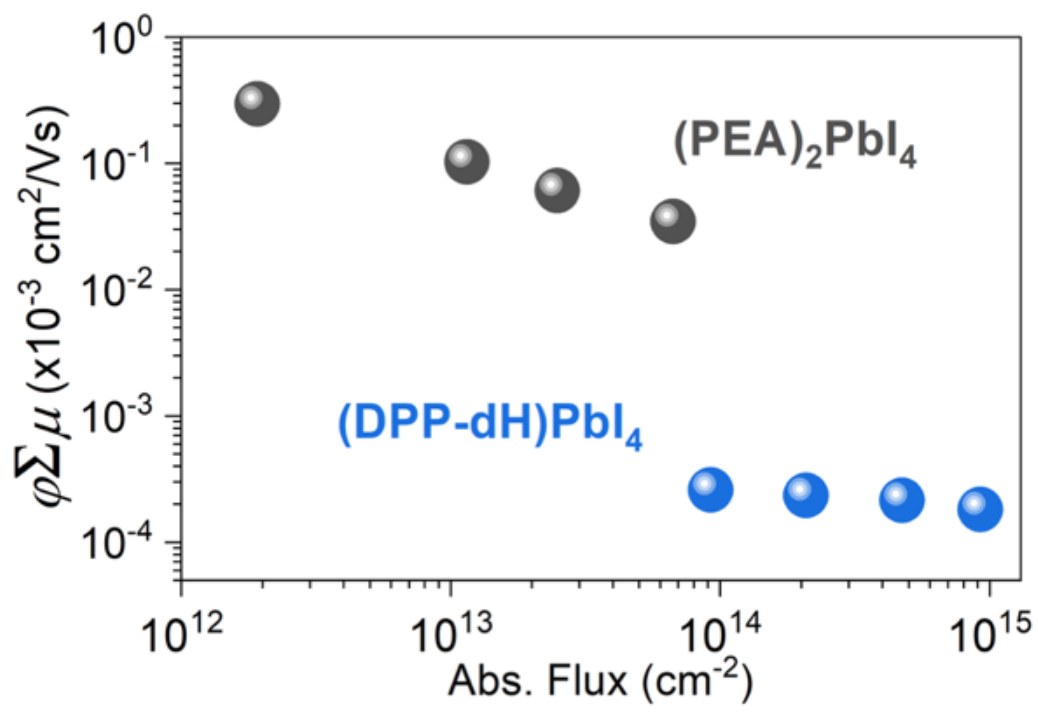

Figure S7. The calculated peak sum mobilities ($\varphi \sum \mu$) of **(DPP-dH)PbI4** and **(PEA)2PbI4** with respect to absorption flux.

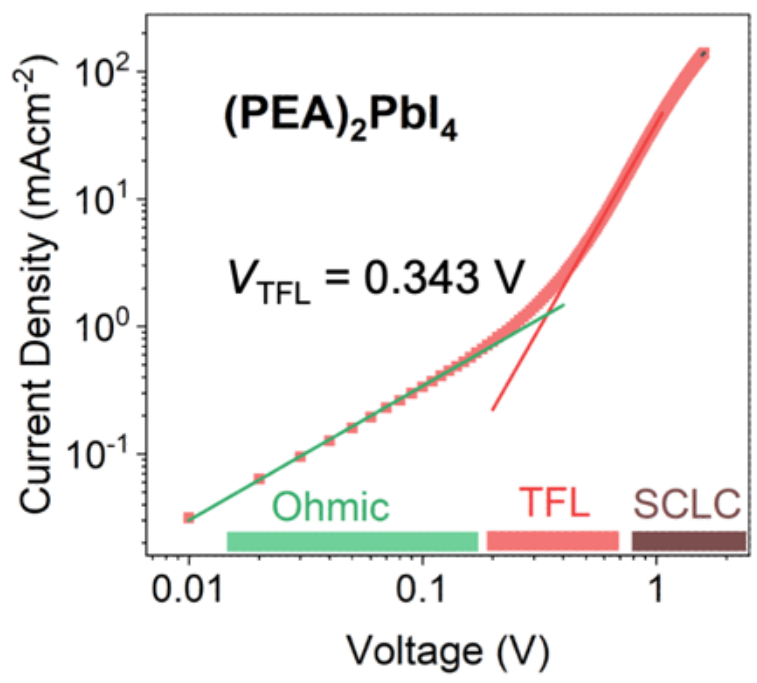

Figure S8. The electron-only SCLC *J*-*V* curve of **(PEA)2PbI4** employing the same architecture as discussed in the main text.

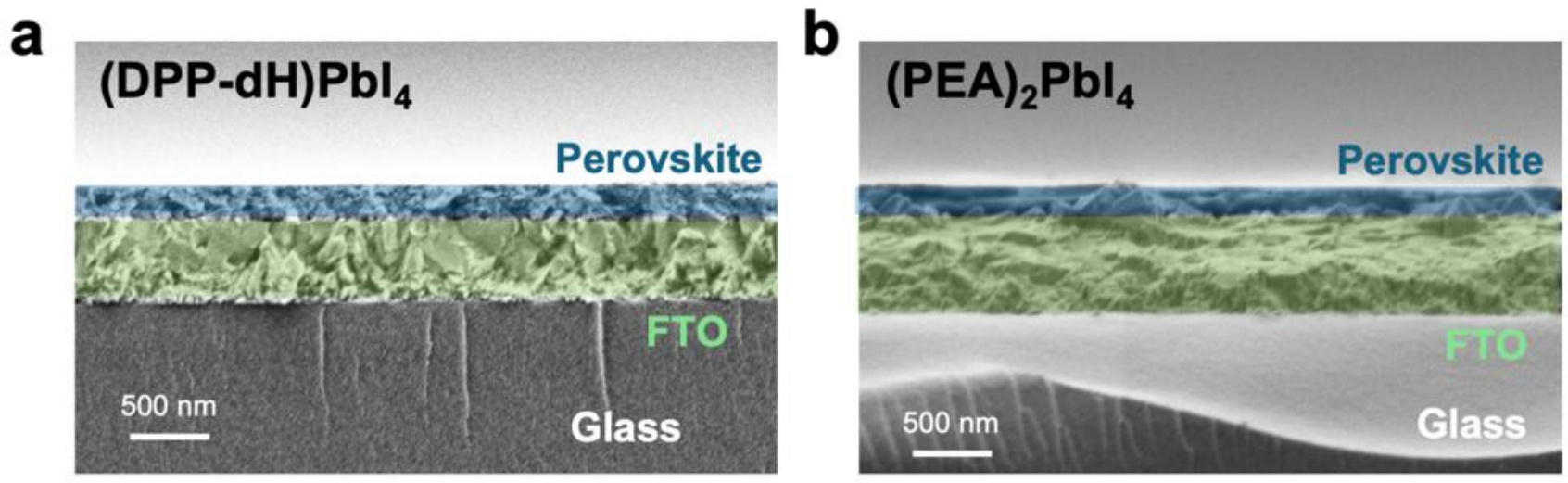

Figure S9. The cross-sectional SEM images of (**a**) **(DPP-dH)PbI$_4$** and (**b**) **(PEA)$_2$PbI$_4$** showing the thickness of the perovskite layer.

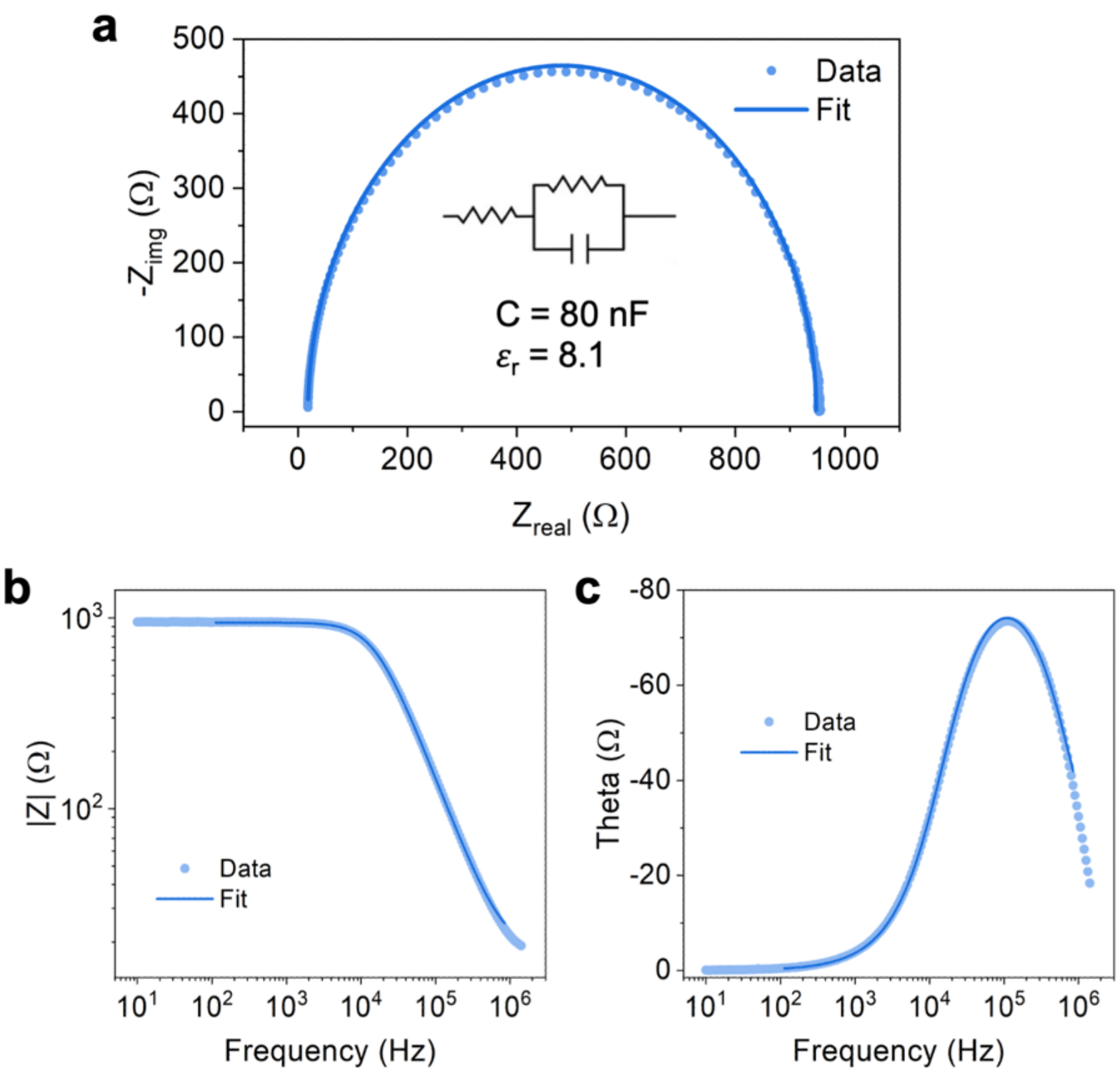

Figure S10. Impedance spectrum of **(DPP-dH)PbI$_4$** and the corresponding fitted curve. The capacitance was extracted from the real and imaginary components of the impedance using the equivalent circuit shown in the inset of (**a**).

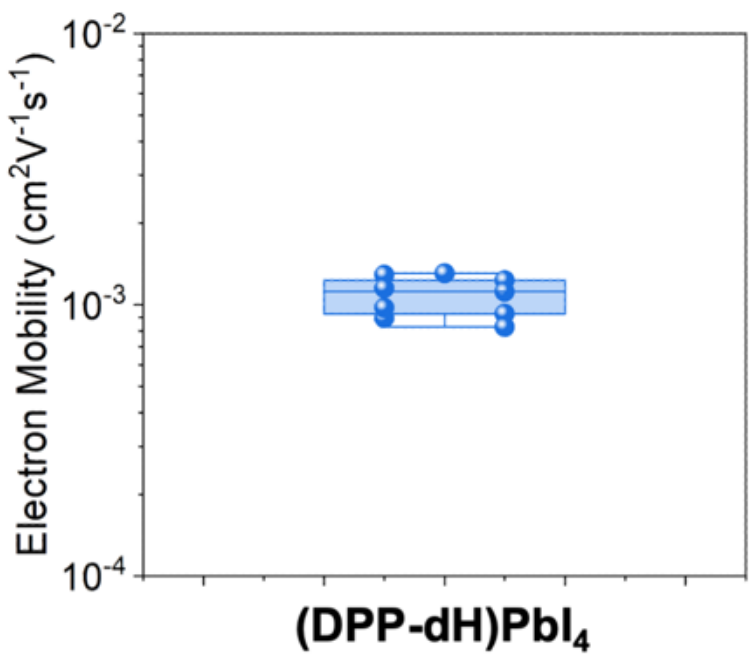

Figure S11. Electron mobilities of **(DPP-dH)PbI**$_4$ extracted from multiple SCLC devices, demonstrating the reproducibility and range of measured mobilities.

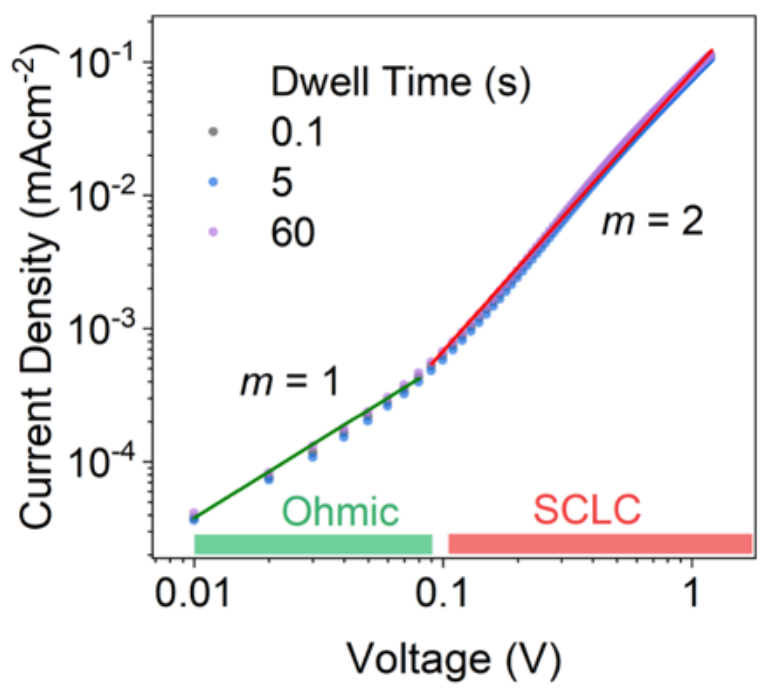

Figure S12. The electron-only 50 ms voltage pulsed-SCLC *J-V* curve of **(DPP-dH)PbI**$_4$ with different dwell time.

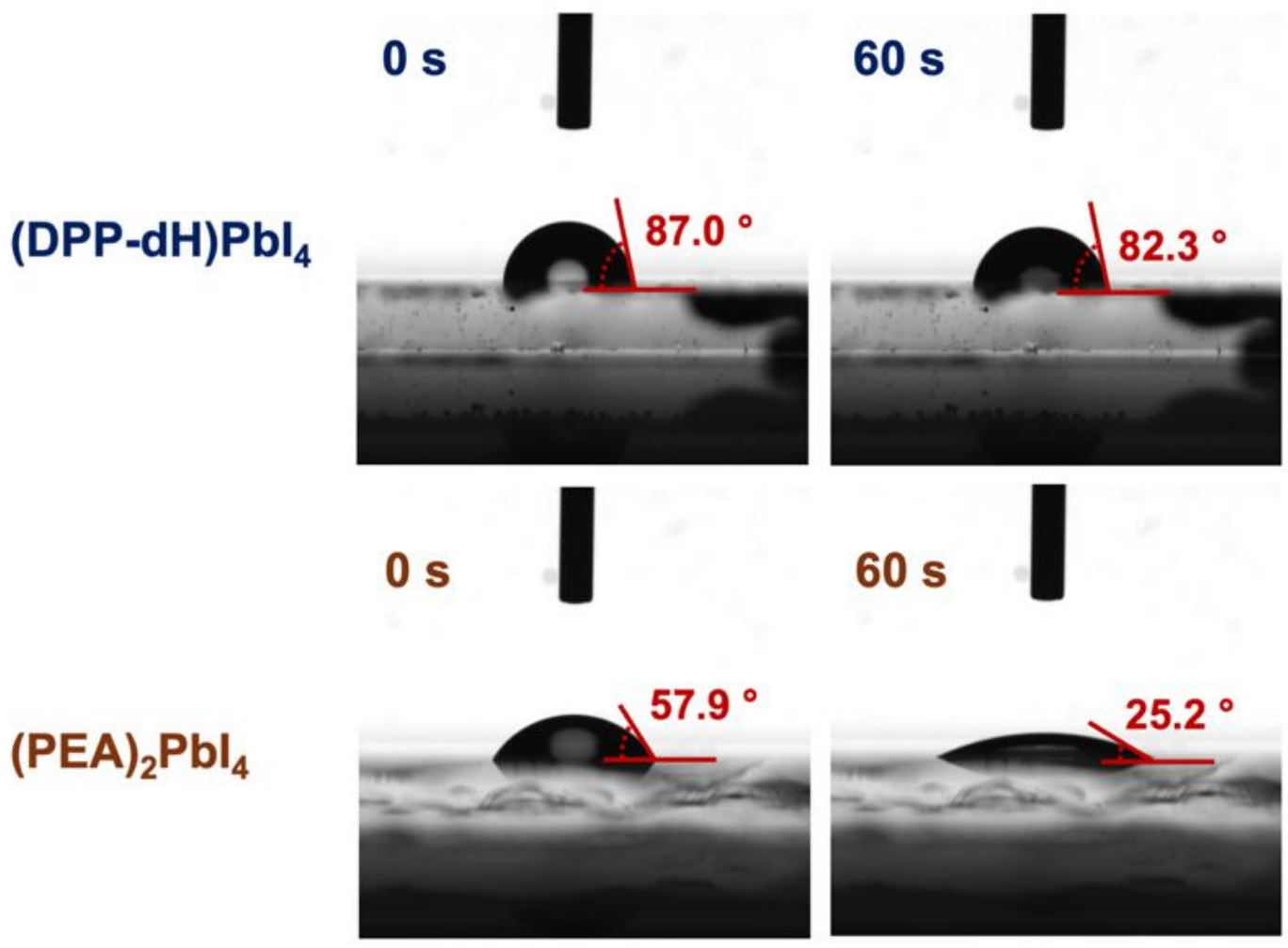

Figure S13. Water contact angle measurements of **(DPP-dH)PbI**$_4$ and **(PEA)**$_2$**PbI**$_4$ films immediately after contact (0 s) and after 60 s. The **(DPP-dH)PbI**$_4$ film exhibits a higher initial contact angle and a smaller decrease over time than **(PEA)**$_2$**PbI**$_4$, indicating enhanced surface hydrophobicity and improved resistance to water degradation.

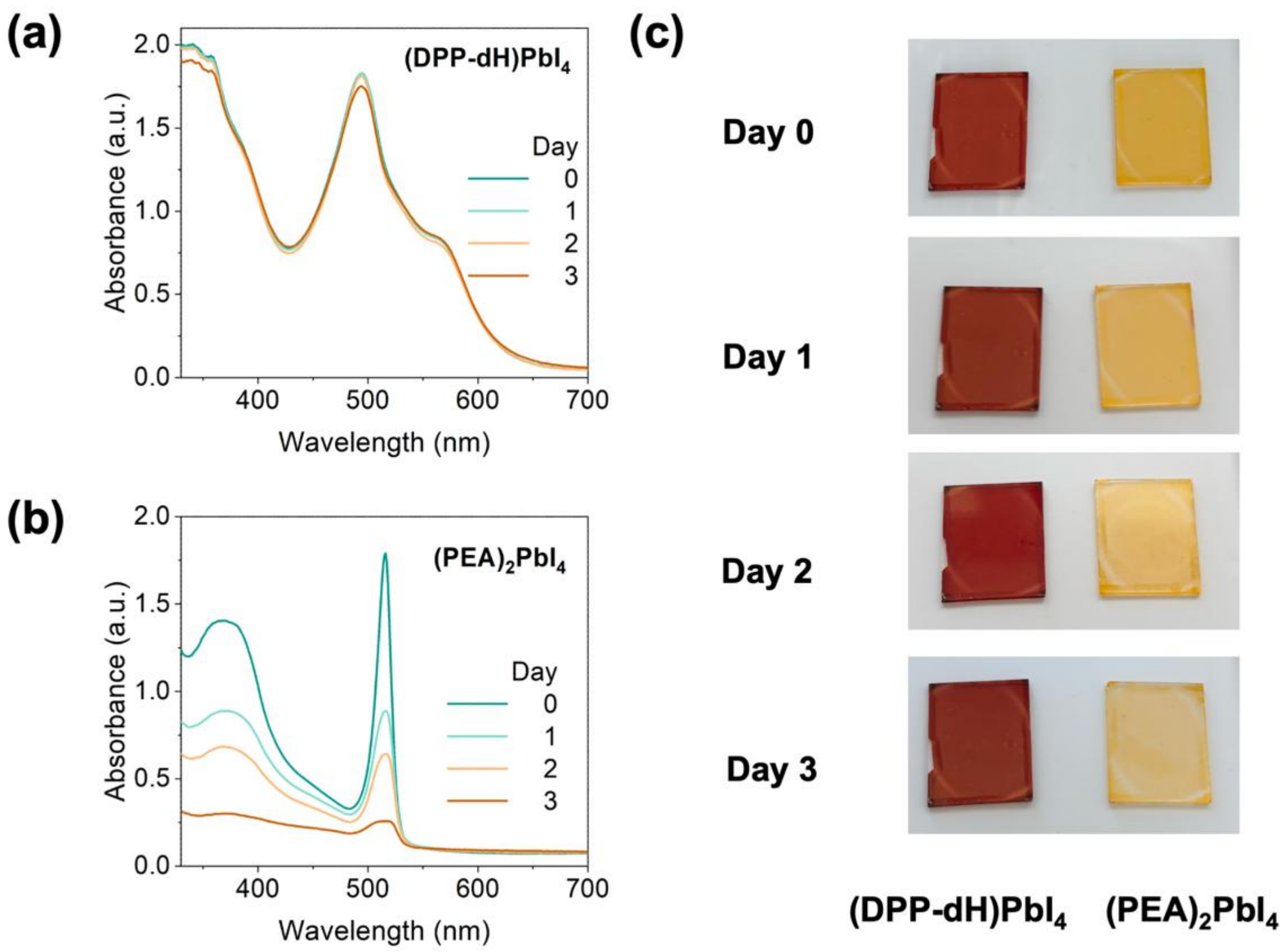

Figure S14. UV-Vis absorption of (**a**) **(DPP-dH)PbI$_4$** and (**b**) **(PEA)$_2$PbI$_4$** after different aging times under 60 ºC and 60% relative humidity. The photograph of the corresponding films aged under same duration are shown in (**c**).

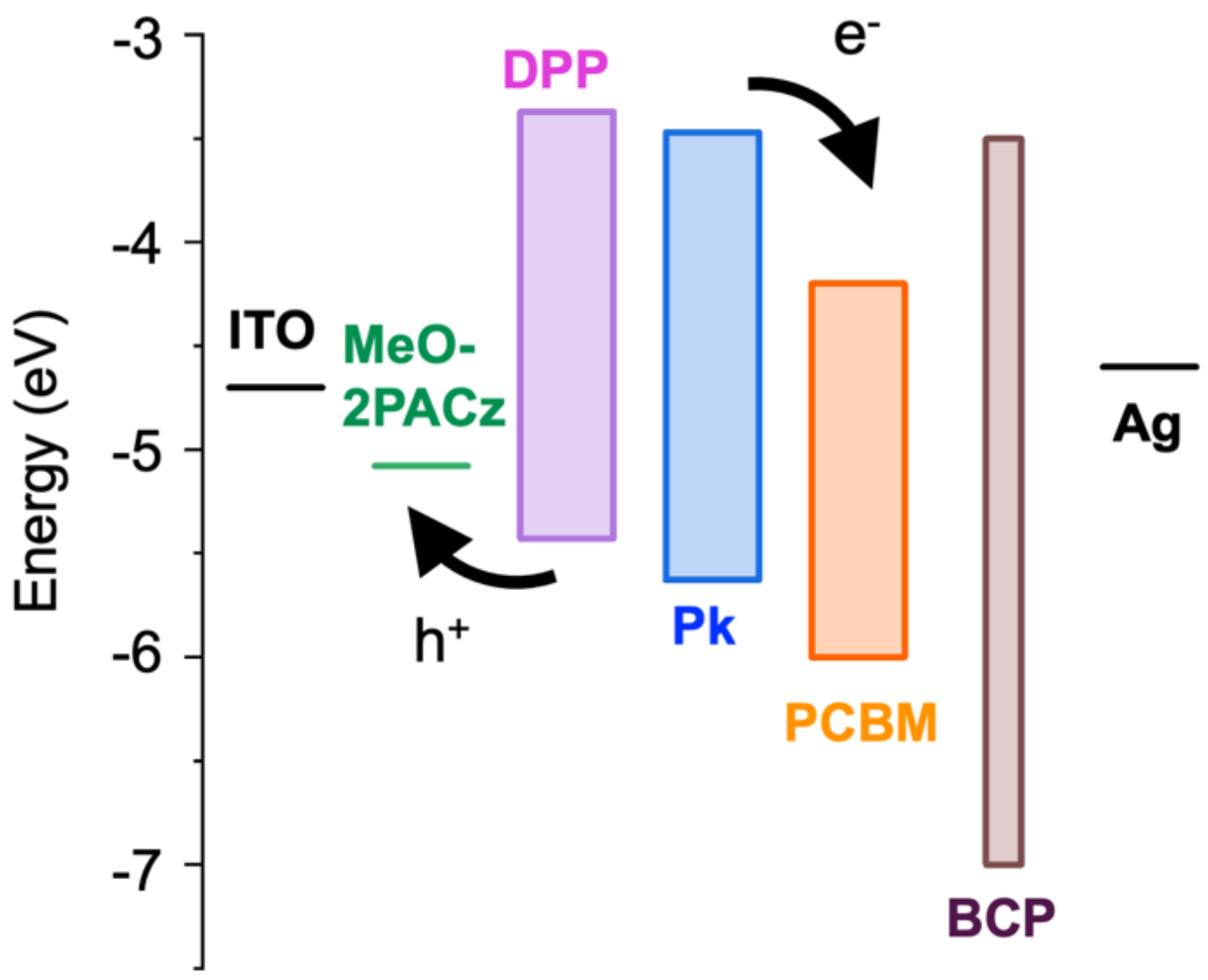

Figure S15. Schematic energy-level alignment of the p-i-n solar cell device incorporating **(DPP-dH)PbI$_4$**.

Table S1. The average PCE, J_{SC}, V_{OC} and FF of **(DPP-dH)PbI4** and **(PEA)2PbI4** over six different devices.

Average	(DPP-dH)PbI$_4$	(PEA)$_2$PbI$_4$
PCE (%)	0.21	0.037
J_{SC} (mA/cm^{-2})	0.67	0.42
V_{OC} (V)	0.60	0.20
FF (%)	55.68	28.35

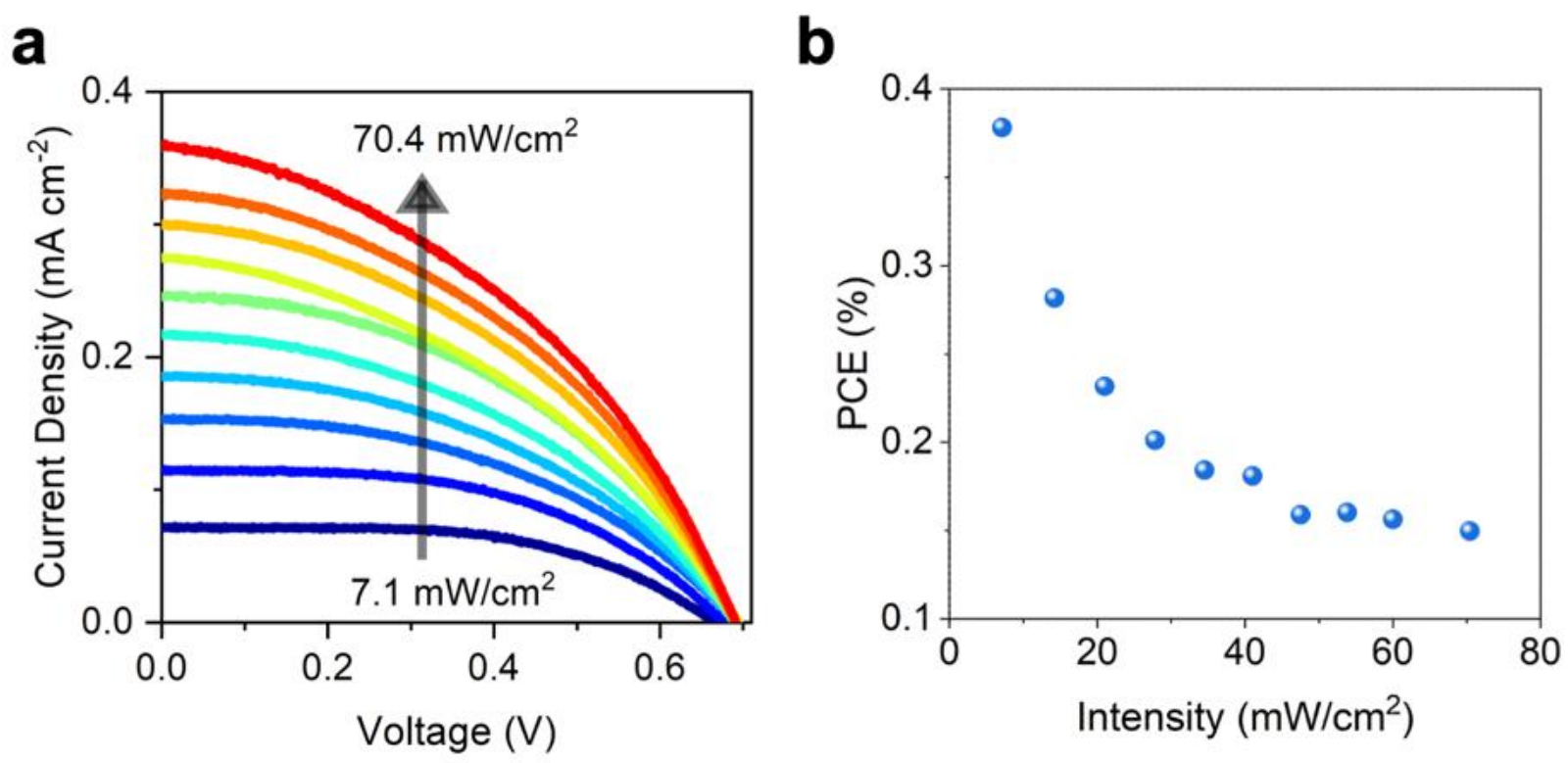

Figure S16. The (**a**) *J-V* curves of **(DPP-dH)PbI4** with different excitation intensities and (**b**) the corresponding PCEs with respect to intensities.

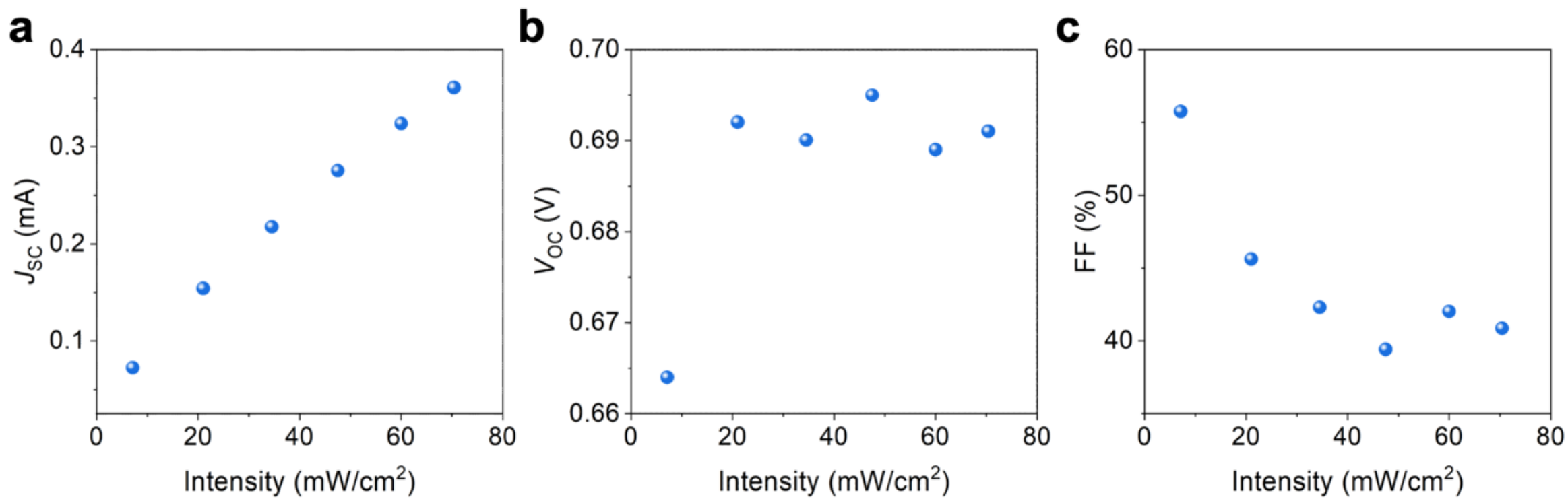

Figure S17. The (**a**) J_{SC}, (**b**) V_{OC}, and (**c**) FF of **(DPP-dH)PbI4** with different excitation intensities.

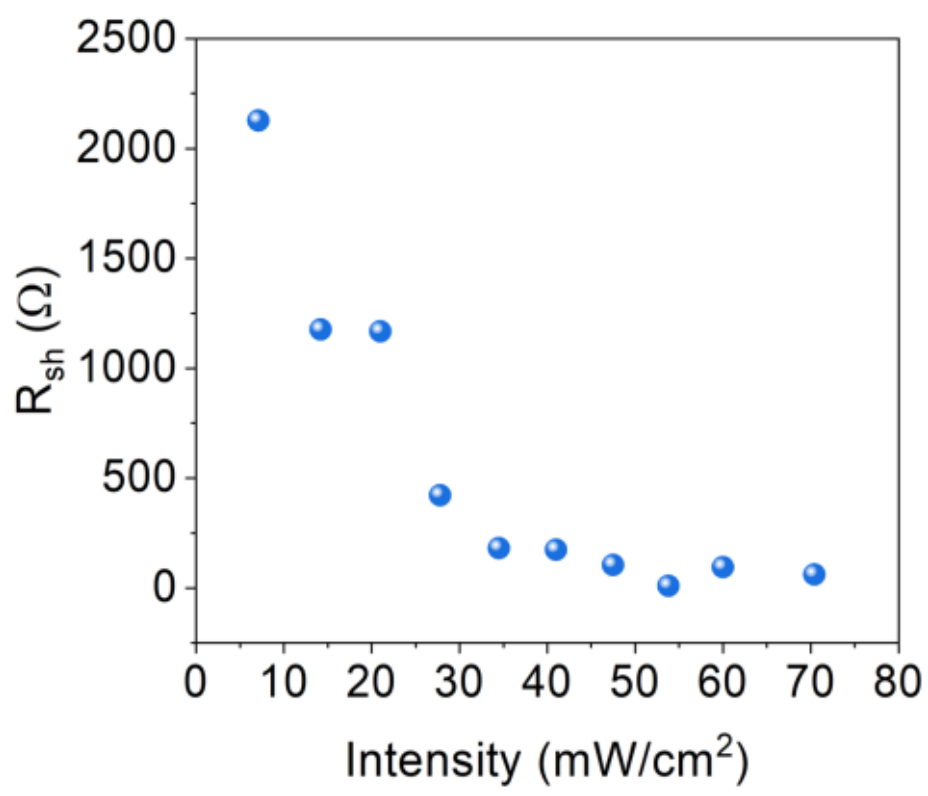

Figure S18. The extracted shunt resistance, R_{sh}, from the *J*-*V* curves in Figure 4c.

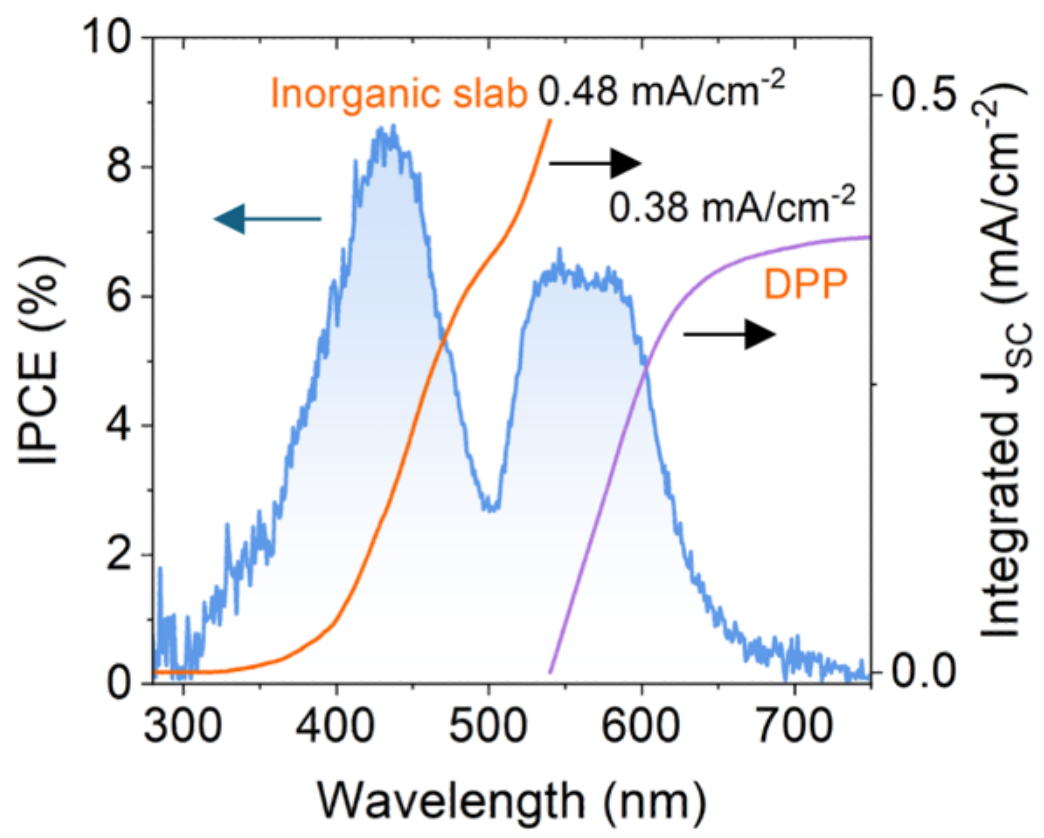

Figure S19. The IPCE curves of **(DPP-dH)PbI4** and the corresponding integrated J_{SC} curves contributed from the inorganic slabs and DPP-dH^{2+} absorption.

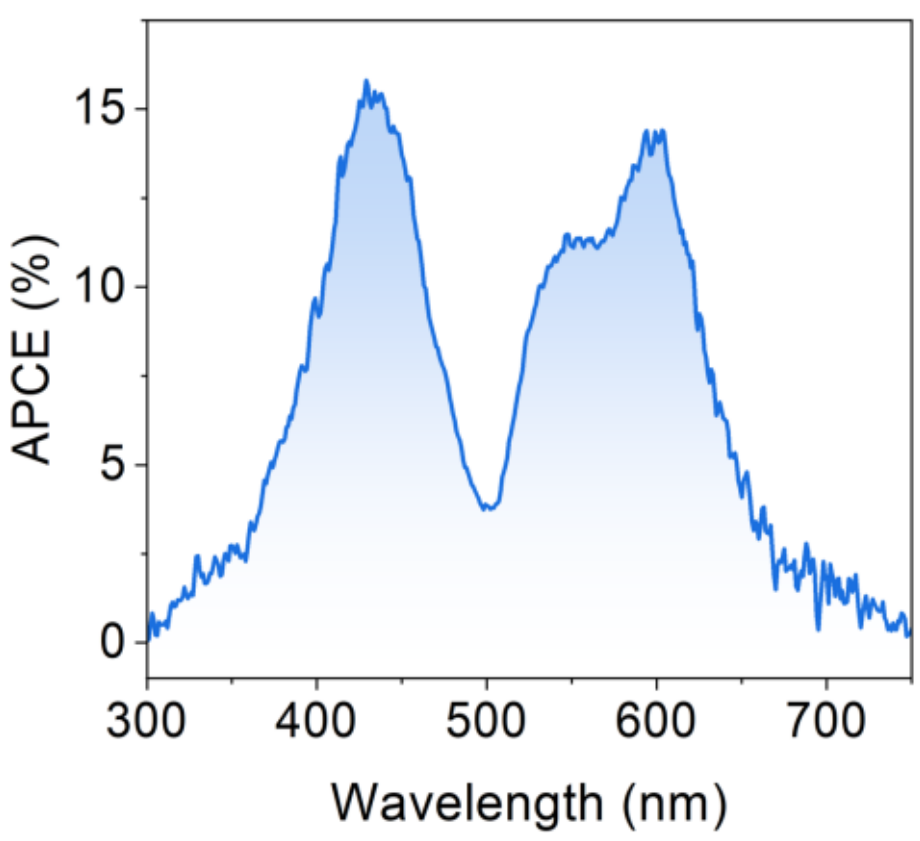

Figure S20. The absorbed photon-to-current efficiency (APCE) of the **(DPP-dH)PbI4**-based device (from Figure 4d).

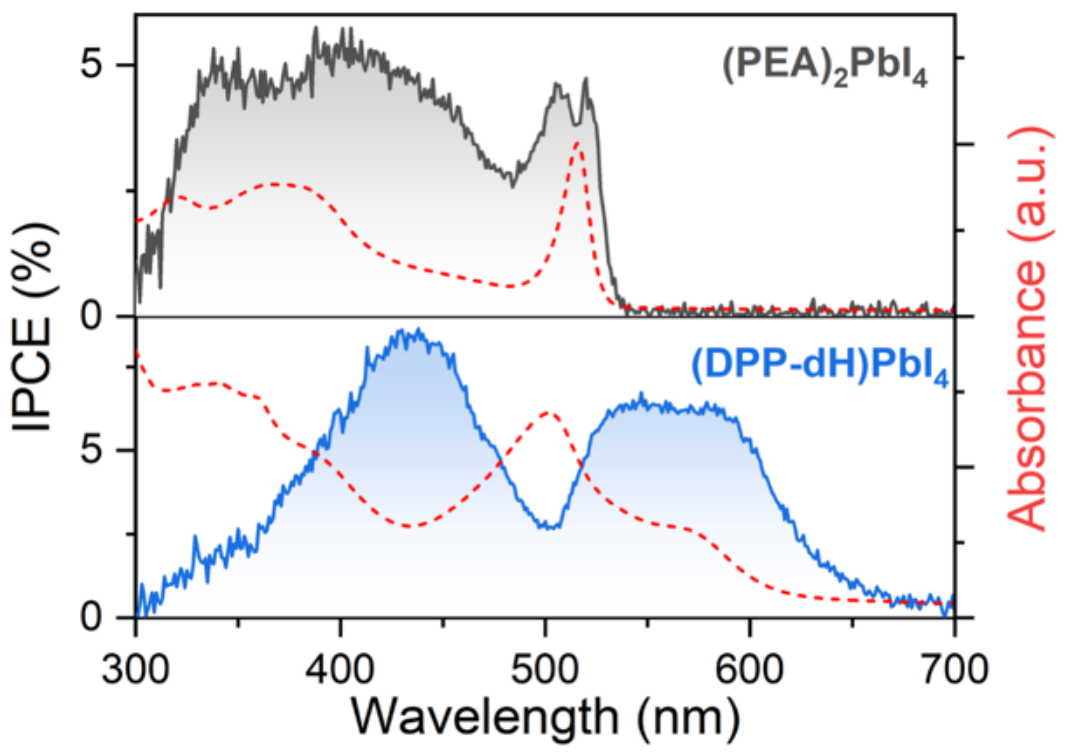

Figure S21. The comparison between the IPCE and absorption spectra of **(PEA)$_2$PbI$_4$** and **(DPP-dH)PbI$_4$**.

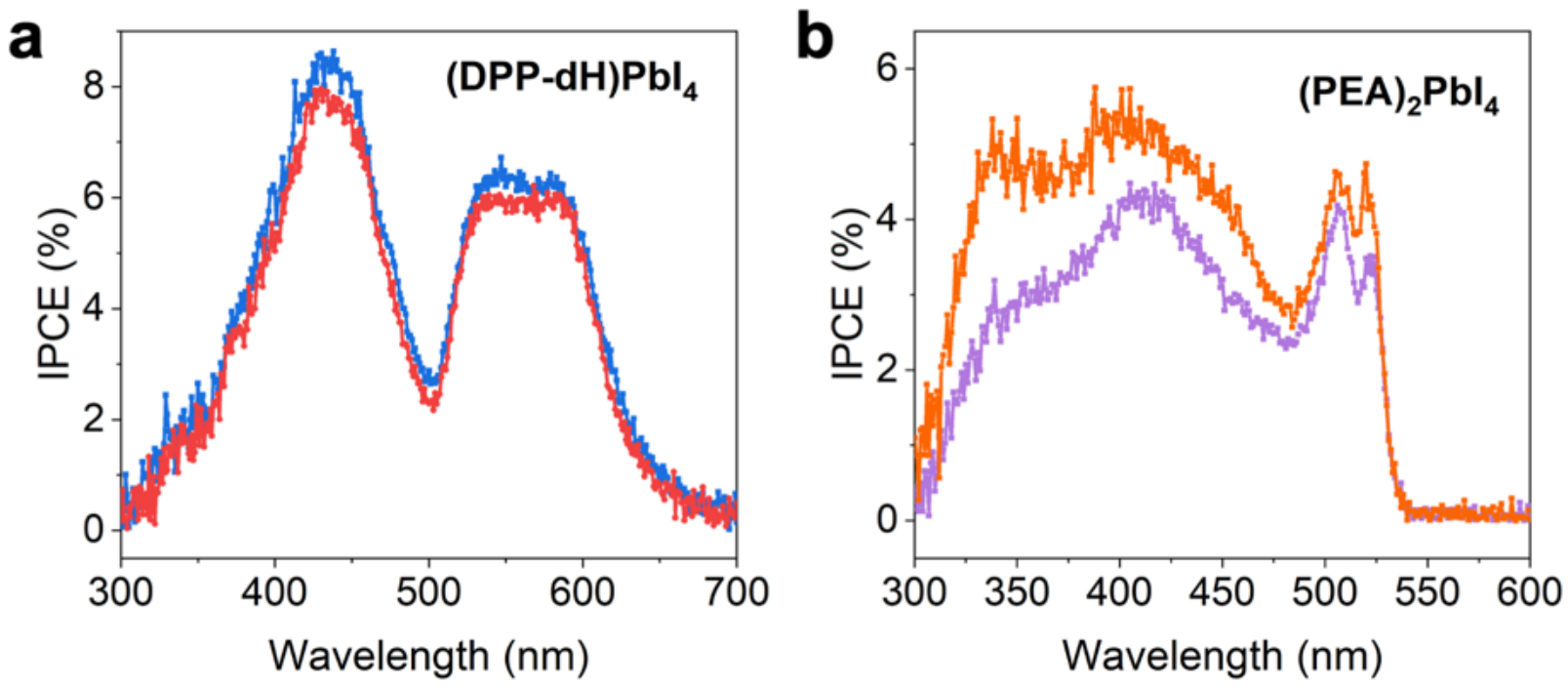

Figure S22. The IPCE curves of (**a**) **(DPP-dH)PbI$_4$** and (**b**) **(PEA)$_2$PbI$_4$** over repeated measurements on different devices.

4. Supplementary Note

Consideration of Alternative Mechanisms for the TA Dynamics of (DPP-dH)PbI$_4$

In the main text, based on the transient absorption (TA) dynamics, we attribute (i) the prolonged photoinduced absorption (PA) signal of the organic spacer following excitation of the inorganic slab (Figures 2c,d) and (ii) a delayed rise of the perovskite ground-state bleach (GSB) upon selective excitation of the organic spacer (Figures 2e,f) of **(DPP-dH)PbI$_4$** to a bidirectional interfacial charge-transfer mechanism. We also considered two alternative interpretations of the observed dynamics: (i) energy transfer (e.g. FRET) between the inorganic slab and the organic spacer and (ii) charge carrier trapping within the inorganic slabs. However, as discussed below, we find that neither mechanism satisfactorily explains the experimental observations.

First, energy transfer between the inorganic slab and the organic spacer would be expected to populate the spacer's excited state and manifest as sensitized photoluminescence. Instead, the photoluminescence of **(DPP-dH)PbI$_4$** is quenched relative to the neat (DPP-dH)I_2 ligand film (Figure S3), and the PA signal is markedly longer-lived than in the neat ligand (~50% of the

population in the perovskite vs. ~7% in the neat ligand; Figure 2d). These two observations are inconsistent with excited-state population via energy transfer, and instead consistent with a non-radiative charge-separation pathway.

Moreover, existence of traps states within the inorganic slab would also produce a long-lived spectral features without charge transfer to the organic component. However, such a process would not be expected to depend strongly on which component is selectively excited. Nonetheless, we observe a pronounced excitation-wavelength dependence: the GSB of **(DPP-dH)PbI$_4$** rises immediately under 400 nm (perovskite-selective) excitation but only after a ca. 50 ps delay under 620 nm (spacer-selective) excitation (Figures 2c,f). This dependence is the expected characteristics of a sequential process where photoexcitation of the spacer followed by charge transfer into the inorganic slab, rather than of intrinsic trapping within the inorganic component alone.

Taken together, these observations are difficult to reconcile with energy transfer or trapping, but are consistent with the proposed bidirectional charge-transfer mechanism.

5. References

[1] A. B. Tamayo, M. Tantiwiwat, B. Walker, T.-Q. Nguyen, *J. Phys. Chem. C* **2008**, *112*, 15543.

[2] E. Zhou, Q. Wei, S. Yamakawa, Y. Zhang, K. Tajima, C. Yang, K. Hashimoto, *Macromolecules* **2010**, *43*, 821.

[3] C. R. Kagan, D. B. Mitzi, C. D. Dimitrakopoulos, *Science* **1999**, *286*, 945.

[4] M. Saliba, J.-P. Correa-Baena, C. M. Wolff, M. Stolterfoht, N. Phung, S. Albrecht, D. Neher, A. Abate, *Chem. Mater.* **2018**, *30*, 4193.

[5] Y. Zhong, M. Hufnagel, M. Thelakkat, C. Li, S. Huettner, *Advanced Functional Materials* **2020**, *30*, 1908920.

[6] P. Ferdowsi, S.-J. Kim, T.-D. Nguyen, J.-Y. Seo, J.-H. Yum, K. Sivula, *J. Mater. Chem. A* **2024**, *12*, 15837.

[7] T. J. Savenije, A. J. Ferguson, N. Kopidakis, G. Rumbles, *J. Phys. Chem. C* **2013**, *117*, 24085.

[8] M. Schleuning, On the Carrier Separation, Dynamics and Optical Properties of Metal Oxide Photoelectrodes, Technische Universität Berlin, **2023**.

[9] V. M. Le Corre, E. A. Duijnstee, O. El Tambouli, J. M. Ball, H. J. Snaith, J. Lim, L. J. A. Koster, *ACS Energy Lett.* **2021**, *6*, 1087.

[10] J. A. Röhr, T. Kirchartz, J. Nelson, *J. Phys. Condens. Matter* **2017**, *29*, 205901.

6. NMR Spectra

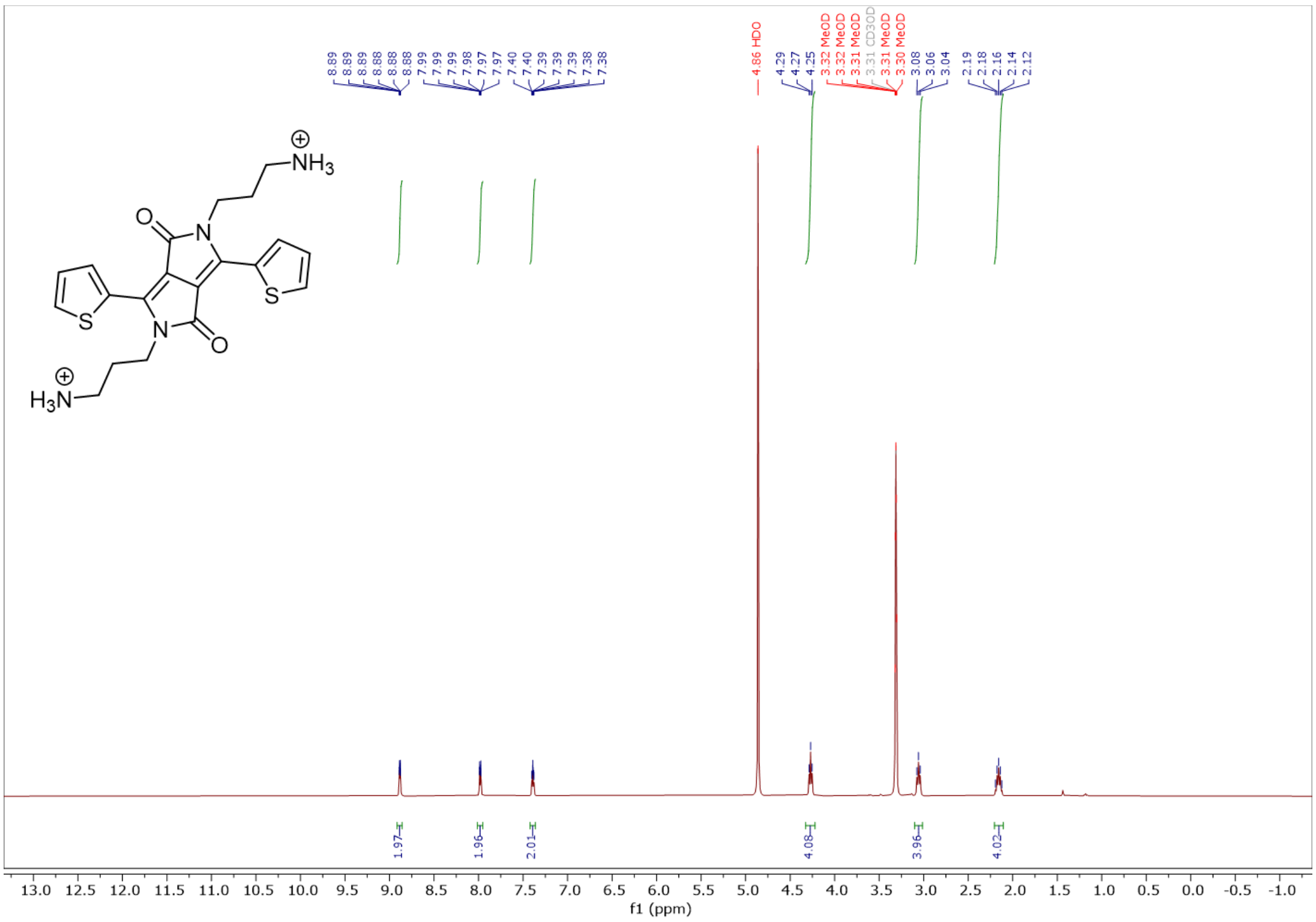

Figure 23. ^{1}H NMR of (DPP-dP)I_2 (MeOD, 400 MHz).

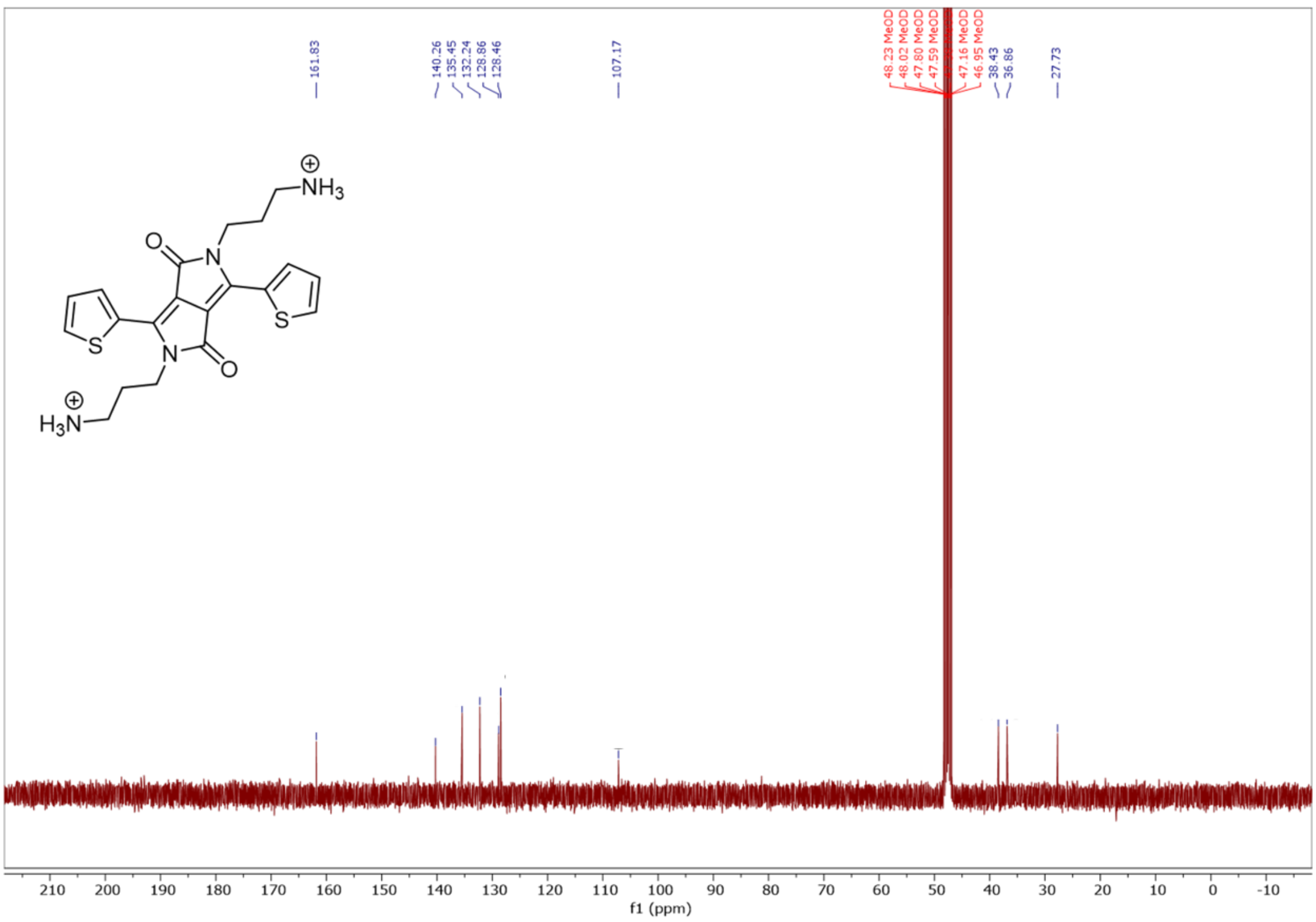

Figure 24. ^{13}C NMR of (DPP-dP)I_2 (MeOD, 400 MHz).

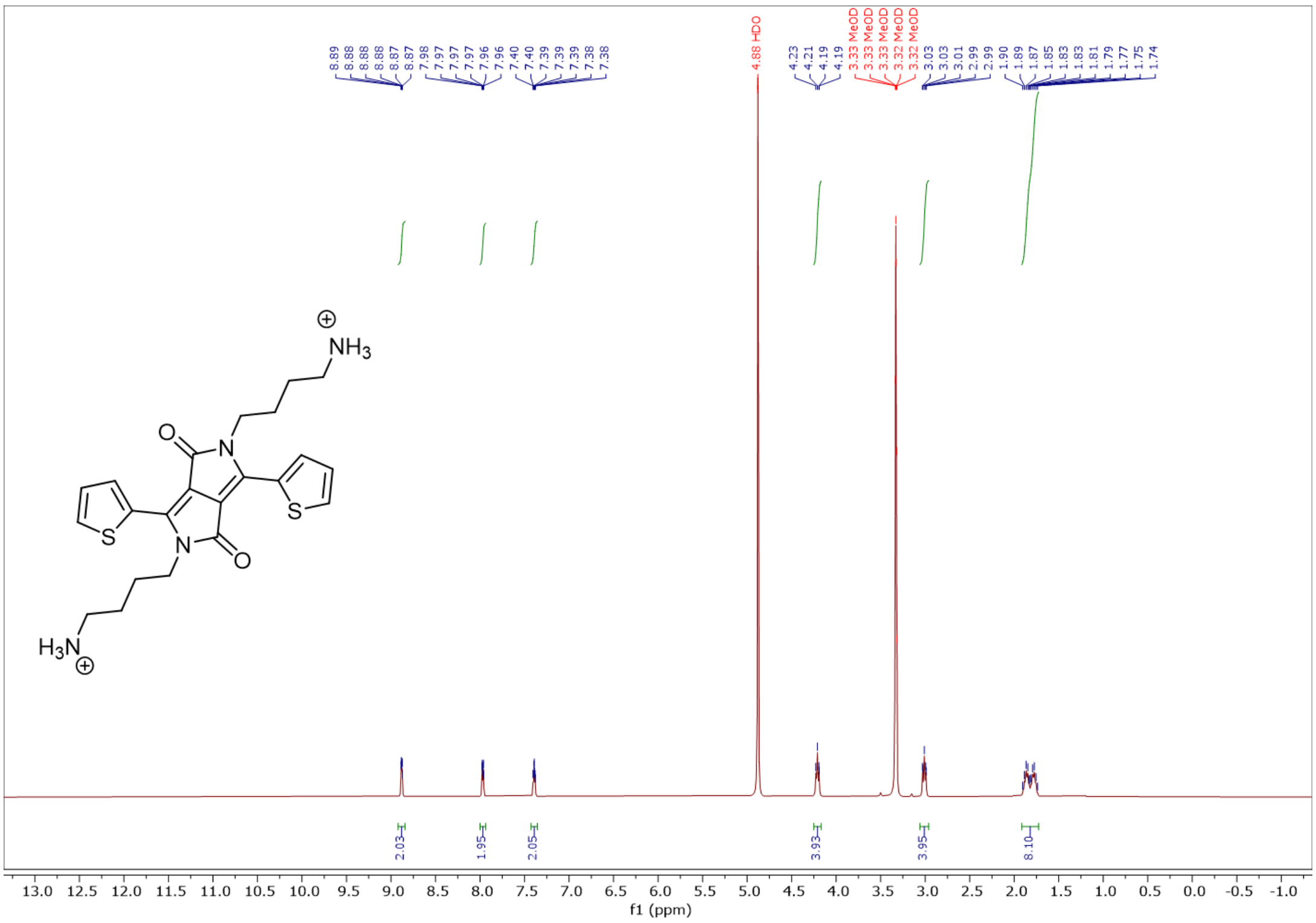

Figure 25. ^{1}H NMR of (DPP-dB)I_2 (MeOD, 400 MHz).

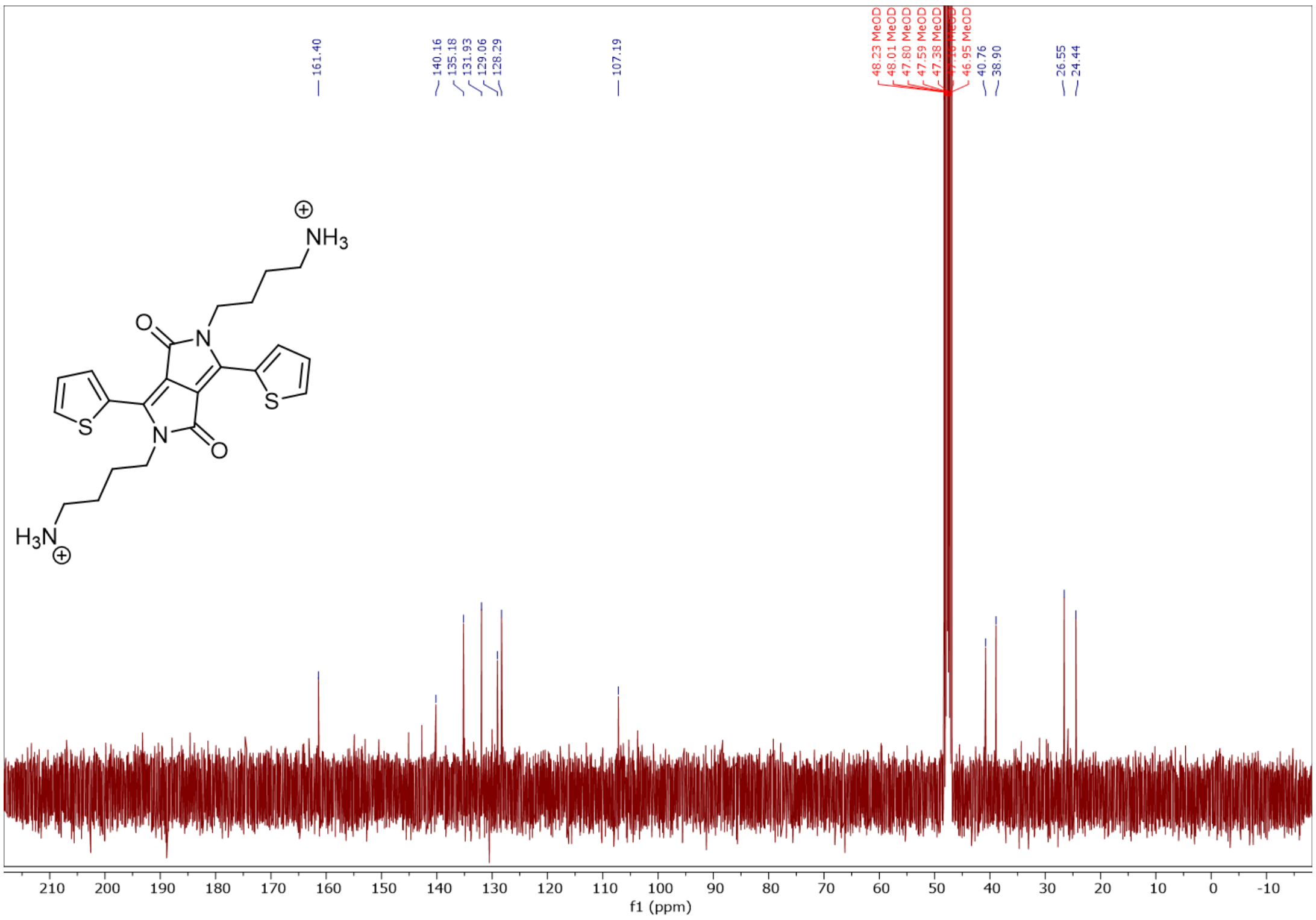

Figure 26. ^{13}C NMR of (DPP-dB)I_2 (MeOD, 400 MHz).

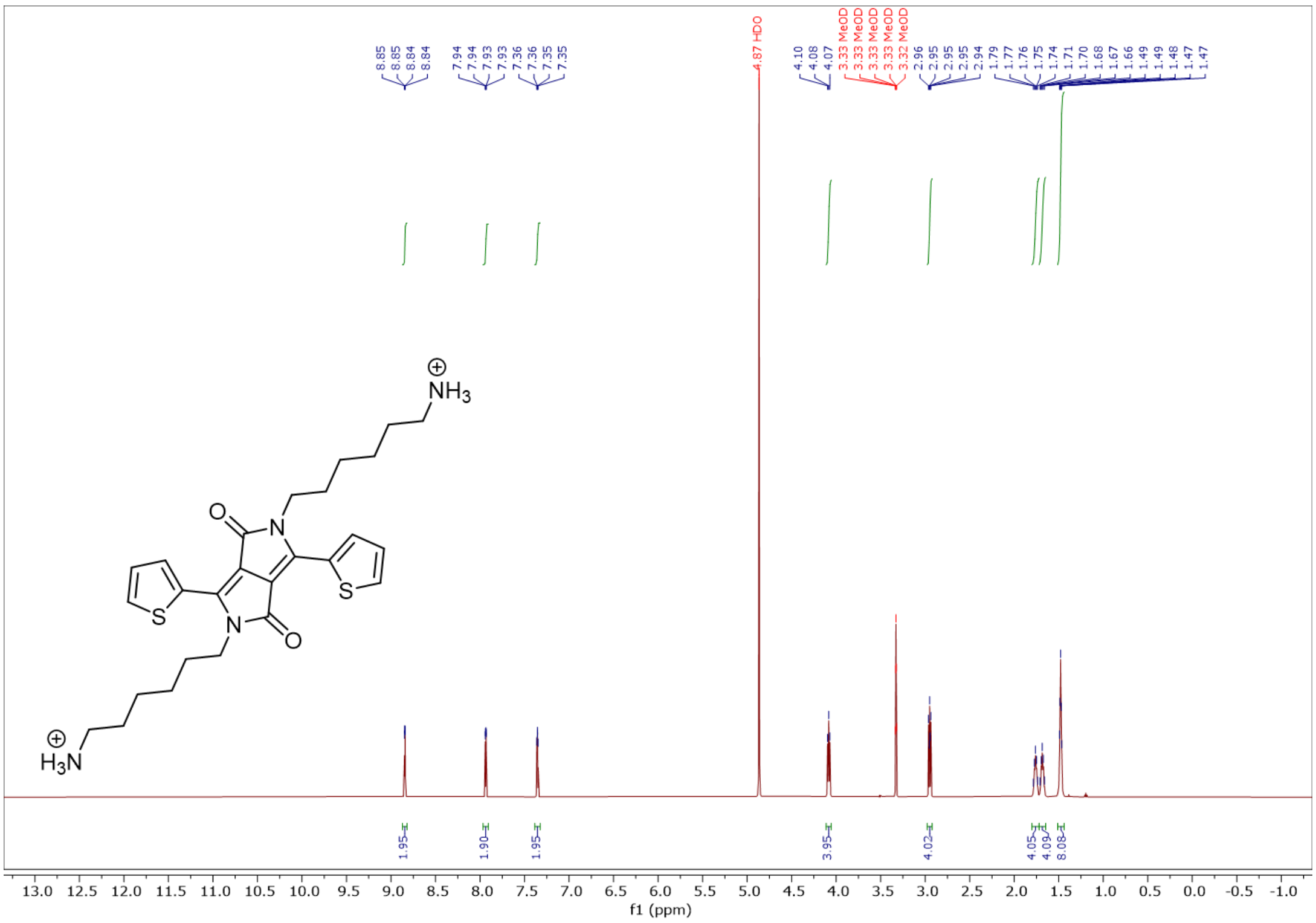

Figure 27. ^{1}H NMR of (DPP-dH)I_2 (MeOD, 400 MHz).

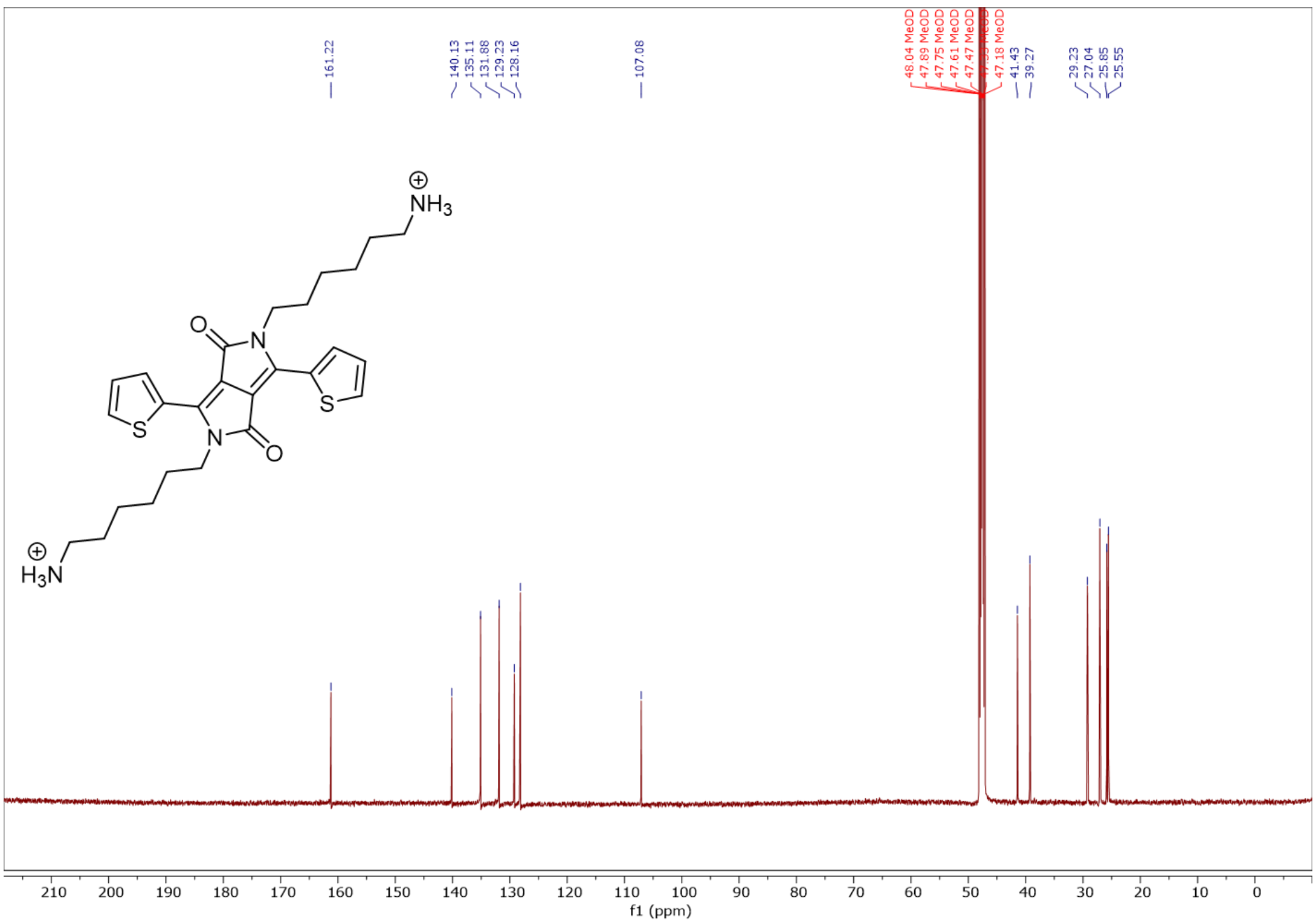

Figure 28. ^{13}C NMR of (DPP-dH)I_2 (MeOD, 400 MHz).
